# Magnetic coupling transforms random snapping into ordered sequences in soft metamaterials

**DOI:** 10.1126/sciadv.aec3182

**Published:** 2026-03-20

**Authors:** Haoze Sun, Gabriel Alkuino, Yinding Chi, Yevhen Zabila, Haitao Qing, Denys Makarov, Teng Zhang, Jie Yin

**Affiliations:** ^1^ Department of Mechanical and Aerospace Engineering, North Carolina State University, Raleigh, NC 27695, USA.; ^2^ Department of Physics, Syracuse University, Syracuse, NY 13244, USA.; ^3^ BioInspired Syracuse: Institute for Material and Living Systems, Syracuse University, Syracuse, NY 13244, USA.; ^4^ Helmholtz-Zentrum Dresden-Rossendorf e.V., Institute of Ion Beam Physics and Materials Research, 01328 Dresden, Germany.; ^5^ Department of Mechanical and Aerospace Engineering, Syracuse University, Syracuse, NY 13244, USA.

## Abstract

Mechanical metamaterials achieve multistep, programmable responses through sequential deformation driven by snapping instabilities, yet these sequences are typically governed by unavoidable imperfections, resulting in random and uncontrollable behavior. Here, we harness intra- and interlayer magnetic interactions coupled with elasticity to reprogram the ordering of sequential buckling instabilities in kirigami-inspired soft magnetic metamaterials. In single-layer systems, intralayer coupling among magnetized units produces random snapping sequences but generates strongly nonlinear-spiked force-displacement responses with pronounced hysteresis, in contrast to the simultaneous buckling of unmagnetized sheets. In multilayer assemblies, interlayer magnetic interactions trigger chain reaction–like propagation, transforming randomness into robust, directional snapping across structures. This mechanism establishes a paradigm for deterministic, multistep mechanical responses without continuously applied fields and opens avenues for adaptive materials in energy dissipation, waveguiding, reconfigurable soft robotics, and biomedical devices.

## Teaser

Internal magnetic interactions transform random sequential snapping into deterministic ordering in planar soft metamaterials.

## INTRODUCTION

Mechanical metamaterials derive their extraordinary properties from architected unit cells that enable unconventional modes of deformation (*1*, *2*). Under uniform global loading, structures composed of identical unit cells typically deform simultaneously, resulting in limited, single-step, and continuous deformation pathways. By contrast, sequential deformation across unit cells unlocks multistep and programmable responses, enriching deformation modes and expanding the tunability of both static and dynamic mechanical properties (*3*–*5*). Among the mechanisms that enable these sequential responses, buckling instabilities are especially attractive for their ability to trigger abrupt shape changes and rapid snapping (*3*, *6*–*11*). This property has been widely exploited in designing multistable and snapping metamaterials for shape morphing (*12*), energy absorption (*7*, *13*), dynamic wave control (*14*), soft robotics (*15*), mechanical computing (*16*–*18*), and electronic devices (*19*). When a global load exceeds critical thresholds, unit cells may undergo localized snapping in a sequential manner, enabling spatiotemporal phase transformations (*3*, *6*–*10*). Yet, because buckling instabilities are highly sensitive to local imperfections introduced during fabrication, the snapping sequence often emerges randomly rather than deterministically (*3*, *6*), posing a major challenge for the rational design of deterministic reconfigurable metamaterials (*12*).

To address this challenge, two main strategies have emerged: geometric and material-based approaches. The geometric approach modifies unit cell parameters or deliberately introduces imperfections to tune buckling thresholds and direct the snapping order (*7*, *8*, *20*, *21*). The material-based approach varies local properties—such as stiffness, viscoelasticity, or plasticity—to alter unit cell responses (*5*, *9*, *22*–*24*). Compared to geometric approaches, material-based strategies generally offer greater flexibility and adaptability. Building on this idea, active metamaterials composed of stimuli-responsive materials allow for tunable properties and deformation under external stimuli—such as heat, light, moisture, electric or magnetic fields—without altering geometry (*9*, *17*, *22*, *25*). These systems enable stimuli-driven sequential phase transitions and programmable mechanical responses (*9*, *22*).

Despite these advances, most active metamaterials rely solely on external stimuli-structure coupling. In contrast, hard-magnetic soft metamaterials (*26*, *27*), formed by embedding magnetically hard microparticles in elastomeric matrices, offer an additional and largely unexplored degree of freedom: persistent internal magnetic interactions that exist even in the absence of an applied field. Unlike other stimuli-responsive systems, these interactions naturally arise between structural components, potentially guiding deformation pathways in a programmable fashion. Recent studies have begun to probe this effect in planar magnetic metamaterials with embedded permanent magnets, revealing its influence on multistability, phase transitions, and wave propagation (*28*–*32*). However, these investigations have been restricted primarily to planar architecture with in-plane rotations and deformations.

This raises several open questions centrally to advancing the field of soft interactive magnetic metamaterials: (i) How do internal magnetic interactions couple with elasticity to produce complex, buckling-induced out-of-plane deformations in magnetized unit cells, and how can these be programmed to direct snapping sequences? (ii) In multilayer assemblies, how do interlayer magnetic interactions alter deformation pathways and collective snapping behavior? Addressing these questions promises to uncover emergent deformation mechanisms and functionalities, advancing the design of deterministic, reconfigurable soft magnetic metamaterials.

Here, we introduce kirigami-inspired interactive soft planar magnetic metamaterials in which intra- and interlayer magnetic interactions are harnessed to program and control snapping sequences. Unlike classical kirigami metamaterials that undergo simultaneous out-of-plane buckling (*33*), the magnetized counterparts exhibit sequential snapping of pop-up units due to intralayer magnetic interactions, producing highly nonlinear mechanical behaviors with enhanced hysteresis. However, the sequence is sample dependent and inherently random. By stacking layers, interlayer magnetic coupling induces chain reaction–like directional snapping, enabling more deterministic control. Leveraging these effects, we demonstrate the use of internal magnetic interactions to enhance energy absorption, opening distinct avenues for programmable mechanical behavior in soft metamaterials.

## RESULTS

### Sequential snapping by intralayer magnetic interactions

**Fig. 1.**
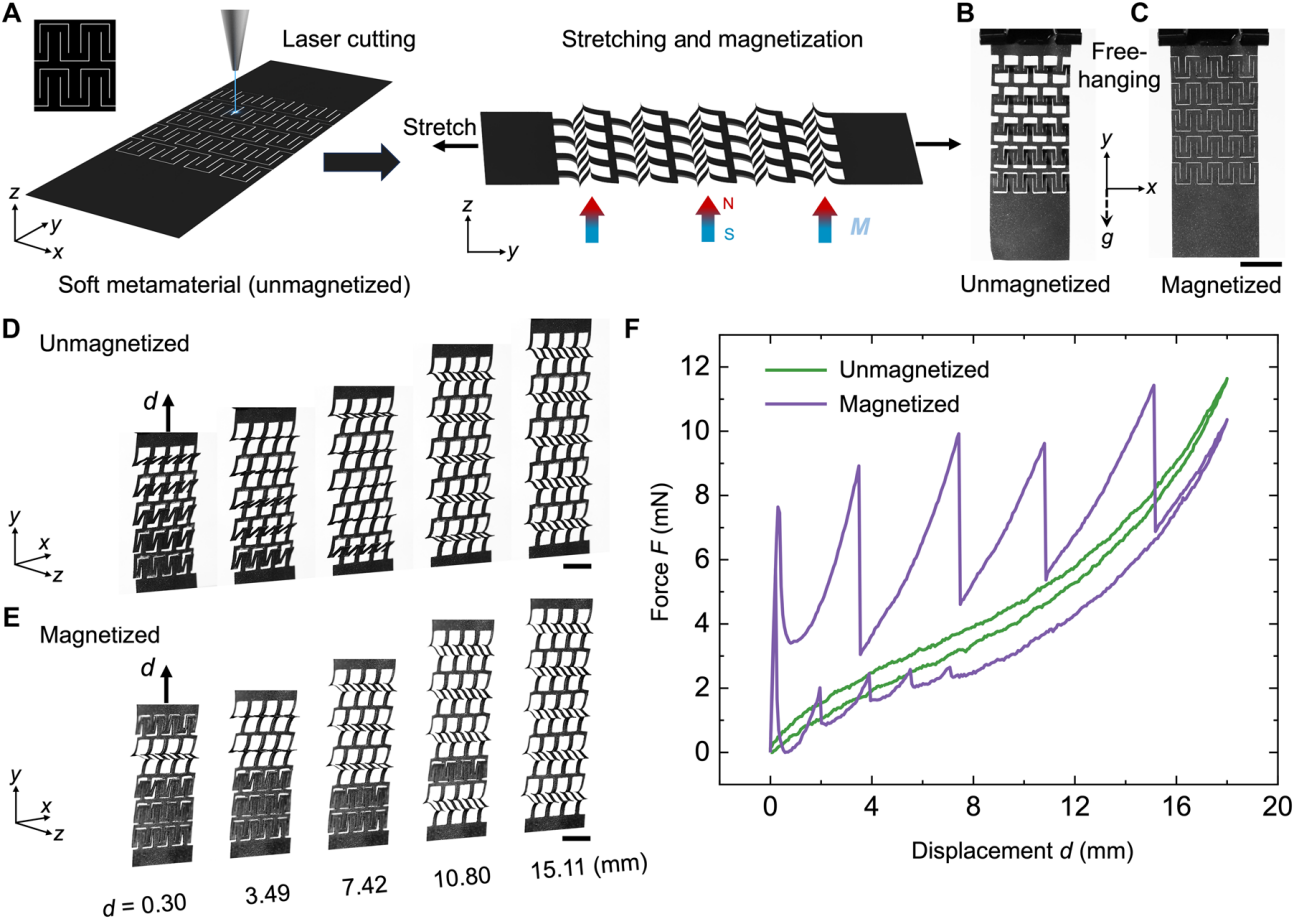
Simultaneous buckling versus sequential snapping in kirigami-inspired soft metamaterials before and after magnetization. (**A**) Schematic of the fabrication process through laser cutting of cut patterns and magnetization (*M* = 1.0 T) under stretch, followed by strain release. (**B** and **C**) Gravity-driven deformation of free-hanging metamaterials before (B) and after magnetization (C). (**D** and **E**) Snapshots of stretch-induced deformation showing simultaneous buckling before magnetization (D) and sequential snapping after magnetization (E). (**F**) Force-displacement curves comparing pre- and postmagnetization responses. Scale bars, 10 mm [(B) to (E)].

Inspired by kirigami cutting strategies (*34*), we designed a planar soft magnetic metamaterial that reconfigure into three-dimensional (3D) via a three-step process (Materials and Methods). A thin elastomeric sheet embedded with hard magnetic particle is first patterned with periodic T-shaped cuts, which buckles out of plane under stretching to form a pop-up lattice (Fig. 1A). The stretched sheet is then magnetized perpendicularly (e.g., *M* = 1.0 T) to establish intralayer magnetic interactions (Fig. 1A). Releasing the stretch returns the sheet to its planar state, yielding an interactive magnetic metamaterial.

The structure exhibits strikingly different mechanical responses before and after magnetization. In the unmagnetized state, the compliant sheet sags under its own weight of 0.75 g, with gravity opening pores via out-of-plane buckling (Fig. 1B). By contrast, the magnetized sheet resists gravity and remains closed, as attractive magnetic interactions keep unit cells in plane (Fig. 1C). Under vertical stretching with a clamped bottom, the unmagnetized sheet undergoes simultaneous buckling: All unit cells pop up together at a small strain (Fig. 1D). This intrinsic simultaneous buckling instability, confirmed by finite element analysis (FEA) simulations (fig. S1), produces a monotonic force-displacement (*F*-*d*) curve with low hysteresis (Fig. 1F). In sharp contrast, the magnetized sample snaps sequentially: As the stretch increases, rows of the unit cells pop up one by one. The first snapping occurs at a displacement of 0.3 mm with a critical buckling force of 7.8 mN (Fig. 1E and movie S1), followed by successive force drops and sharp spikes in the *F*-*d* curve. This sequential response increases hysteresis close to fourfold and enhances initial stiffness by ~18× relative to the unmagnetized case (Fig. 1E). Similar sequential snapping is observed in magnetized metamaterials with curved cut motifs (fig. S2 and movie S2).

**Fig. 2.**
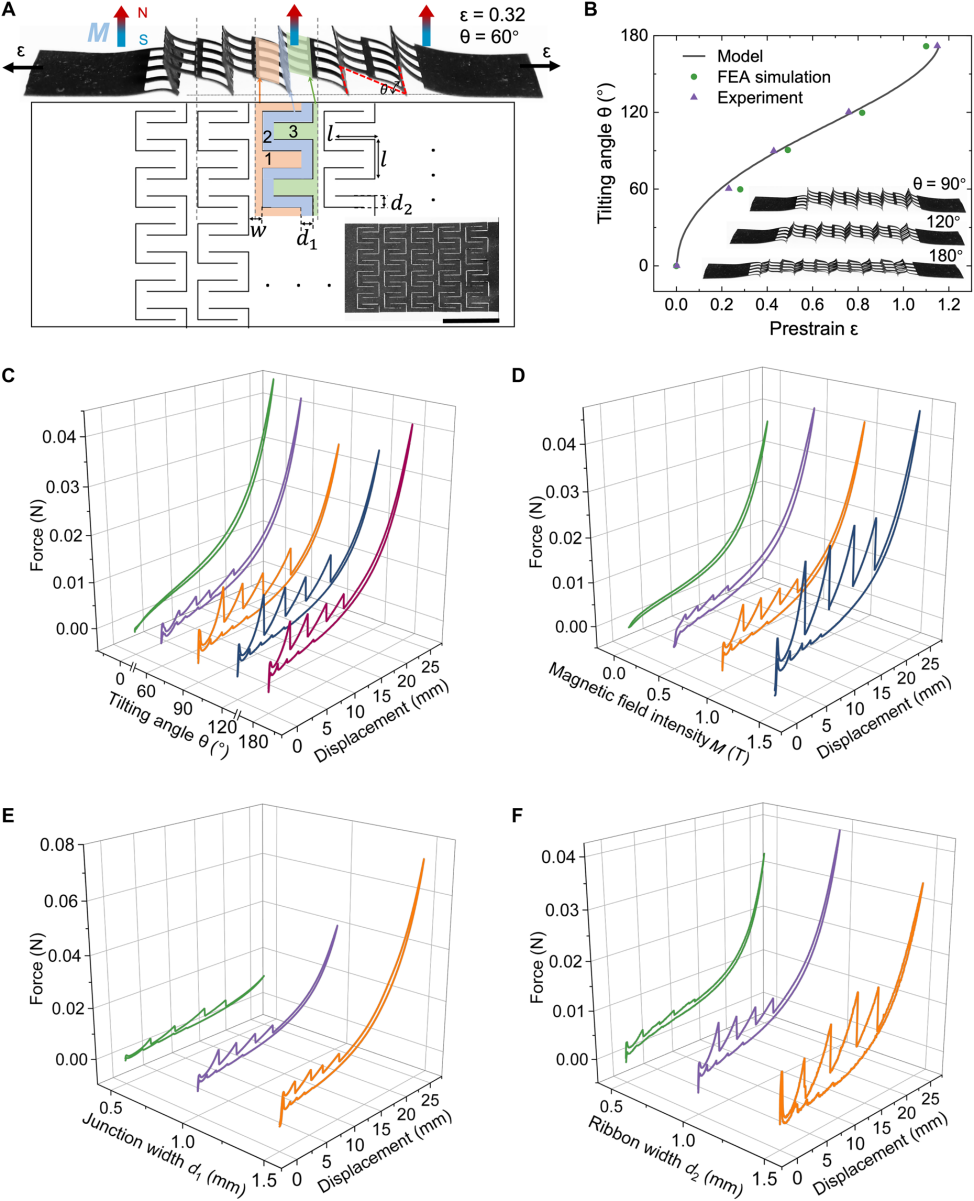
Effects of magnetization and unit-cell geometry on force-displacement (*F*-*d*) responses. (**A**) Schematic of design parameters, including unit geometry (cut length *l*, spacing *w*, junction width *d*_1_, and ribbon width *d*_2_) and magnetization parameters (pre-strain ε, tilting angle θ, and magnetic field intensity **M** applied perpendicular to the stretching plane). Inset: sample image before stretching. Highlighted zones 1 to 3 represent the magnetic interactions between two adjacent pop-up rows with zone 2 tilting out of plane. (**B**) Dependence of tilting angle θ on prestrain ε from theory, simulation, and experiments (*w* = 1 mm, *l* = 3.5 mm). Insets show the stretched tilting states at selected θ. (**C**) *F*-*d* curves as a function of θ (*M* = 1.5 T, *d*_1_ = *d*_2_ = 1 mm). (**D**) *F*-*d* curves as a function of *M* (θ = 90°, *d*_1_ = *d*_2_ = 1 mm). (**E**) *F*-*d* curves as a function of *d*_1_ (θ = 90°, *M* = 1.5 T, *d*_2_ = 1 mm). (**F**) *F*-*d* curves as a function of *d*_2_ (θ = 90°, *M* = 1.5 T, *d*_2_ = 1 mm).

The emergent sequential snapping, enlarged hysteresis, and increased stiffness arise from the interplay between magnetic interactions (heighted zones 1 to 3 in Fig. 2A) and elasticity, tunable through the magnetization process and geometry (Fig. 2, A and B). We first examine the effect of the tilting angle θ of the pop-up units during magnetization. From geometry

$$\theta ={\text{cos}}^{-1}\left\{-\frac{{\left[\left(w+l\right)\left(\varepsilon +1\right)\right]}^{2}+l^{2}-{\left(2l+w\right)}^{2}}{2\left(w+l\right)\left(\varepsilon +1\right)l}\right\}$$ (1)

where *w* is the unit-cell spacing, *l* is the cut length of the square T-shape (Fig. 2A), and ε is the prestretching strain. For a representative geometry (e.g., *w* = 1 mm and *l* = 3.5 mm), θ increases monotonically from 0° to 180° as ε rises from 0 to 115% (Fig. 2B). Accordingly, unit cells transition from flat and closed to partially open to fully open but nearly flattened configurations (Fig. 2B, inset). This analytical prediction agrees well with both experiments and FEA simulations (Fig. 2B). The simple geometric model is valid only within a moderate-strain regime (0 ≤ ε ≤ 1.14) for an upper bound angle θ of 180°. Beyond this point, the unit cells are fully opened and overstretched, and their deformation mode transitions from rotational opening to global membrane stretching, rendering the model inapplicable.

Since the magnetization field **M** is fixed perpendicular to the stretching plane, the varying titling angle θ under different prestretched states misaligns the unit cells with the field, producing distinct *F*-*d* curves even at the same *M* (*M* = 1.5 T; Fig. 2C). As θ increases from 0° to 180°, sequential snapping emerges, evidenced by five consecutive force drops in the *F*-*d* curves. The effect is most pronounced at θ = 90°, where the unit-cell tilting direction aligns with the magnetization field. In this case, strong intralayer attraction leads to the highest critical buckling forces and hysteresis (Fig. 2C). The first critical buckling force more than doubles (*F*_cr_ ~ 13 mN) compared to θ = 60° (fig. S3A), and hysteresis increases by about 2.5-fold compared to θ = 0° (fig. S4A). In contrast, magnetization at θ = 0° (no prestretch) yields a slightly open structure due to repulsive unit-cell interactions (fig. S1A), producing a continuous *F*-*d* curve without snapping (Fig. 2C), similar to the unmagnetized case despite magnetization. At θ = 180°, despite its nearly flattened configuration during magnetization, the metamaterial still exhibits strong attraction, with *F*_cr_ ~ 10 mN and doubled hysteresis compared to θ = 0° (figs. S3A and S4A). Thus, θ = 90° is chosen for subsequent magnetization. Increasing *M* further amplifies sequential snapping: Raising *M* from 0.5 to 1.5 T doubles the critical buckling force and increases hysteresis by ~2.7× (figs. S3B and S4B).

Geometry further modulates the snapping behavior. For fixed *w* and *l*, the junction width *d*_1_ and the ribbon width *d*_2_ (Fig. 2A) are key parameters. While both influence the snapping response, widening *d*_2_ markedly raises critical buckling forces and hysteresis (Fig. 2, D and E, and fig. S3, C and D and S4, C and D). This arises from the dominated role of ribbon bending in governing deformation.

### Modeling and simulation of sequential snapping

**Fig. 3.**
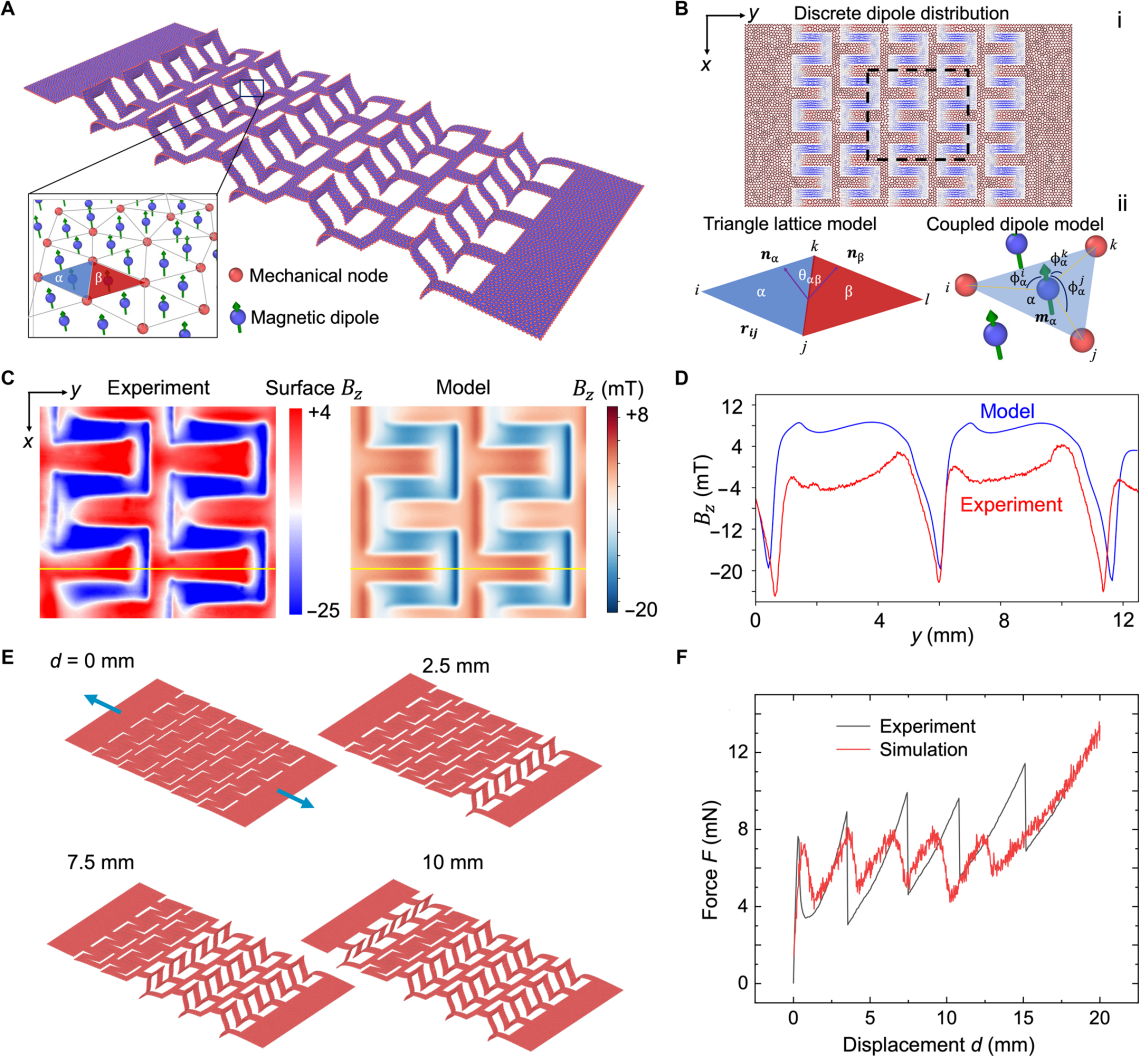
Modeling and simulation of sequential snapping. (**A**) Schematic of the triangular lattice model coupling mechanical nodes (red) at the vertices of the mesh with magnetic dipole nodes at the element centers. (**B**) Simulated discrete dipole distributions after magnetization and strain release. (**C** and **D**) Comparison between the simulated and experimentally measured out-of-plane magnetic field contour (*B_z_*) near the surface (C), and its magnetic field profile along the yellow line in (C), (D). (**E**) Snapshots of simulated sequential snapping at different displacements. (**F**) Comparison between the simulated and experimental force-displacement curves.

To gain mechanistic insight into sequential snapping, we develop a triangular lattice model (*35*–*38*) in LAMMPS (*39*) that couples elastic deformation with magnetic interactions. Each triangular mesh element is represented by three mechanical nodes at its vertices and a dipole node at its centroid (Fig. 3A), enabling the simultaneous capture of elastic deformation and magnetic interactions. The discrete dipole distribution in the fully released state is obtained by replicating the experimental magnetization process at a stretched tilt angle of θ = 90° (Fig. 3B, fig. S5, and the Supplementary Materials) with the out-of-plane magnetic field *B_z_* near the surface shown in Fig. 3C. The simulated dipole distribution is verified by comparing *B_z_* with experiments (fig. S6 and see Materials and Methods for details), which shows good qualitative agreement in the magnetization patterns (Fig. 3C) and the *B_z_* curve profiles along the yellow line (Fig. 3D). The magnetization pattern shows consistent strong attractive interactions between neighboring struts with experiments.

Several models based on discrete differential geometry have been developed to compute the elastic energy of thin sheets (*35*–*38*, *40*, *41*). Here, we use a triangle lattice model (*35*–*38*), in which stretching occurs along edges and bending along dihedrals

$$U_{E}=\frac{\sqrt{3}}{4}\mathrm{Et}\sum_{\text{edge}}{\left(l_{e}-l_{e}^{0}\right)}^{2}+\frac{2}{\sqrt{3}}K\sum_{\text{dihedral}}\left(1-n_{\alpha }\cdot n_{\beta }\right)$$ (2)

where *E* is the Young’s modulus, *t* is the thickness, *K* is the bending stiffness, $l_{e}^{0}$ and $l_{e}$ are the reference and current edge lengths, and $n_{\alpha }$ and $n_{\beta }$ are the normals of the triangles forming the dihedral (Fig. 3B). To couple magnetic interactions, each dipole node is constrained to the shell deformation through a penalty energy

$$\begin{array}{cc} U_{P} & =\sum_{\alpha }\frac{1}{2}k_{c}{\left\|x_{\alpha }-{\bar{x}}_{\alpha }^{\mathrm{ijk}}\right\|}^{2}\\ & +\sum_{\alpha }\frac{1}{2}k_{d}\left[{\left(\phi_{\alpha }^{i}-{\phi_{\alpha }^{i}}^{0}\right)}^{2}+{\left(\phi_{\alpha }^{j}-{\phi_{\alpha }^{j}}^{0}\right)}^{2}+{\left(\phi_{\alpha }^{k}-{\phi_{\alpha }^{k}}^{0}\right)}^{2}\right] \end{array}$$ (3)

where $x_{\alpha }$ is the dipole position, ${\bar{x}}_{\alpha }^{\mathrm{ijk}}$ is the centroid, $k_{c}$ and $k_{d}$ are penalty stiffnesses, and ϕ terms enforce dipole alignment. The dipole-dipole interaction energy follows the standard form (*42*)

$$U_{M}=\sum_{\alpha <\beta }\frac{\mu_{0}}{4\pi r_{\alpha \beta }^{3}}\left[m_{\alpha }\cdot m_{\beta }-3\left(m_{\alpha }\cdot {\hat{r}}_{\alpha \beta }\right)\left(m_{\beta }\cdot {\hat{r}}_{\alpha \beta }\right)\right]$$ (4)

with forces and torques given in the Supplementary Materials. This framework captures the nonlinear, nonlocal coupling between elasticity and magnetism.

The model reproduces the sequential disordered snapping observed experimentally (Fig. 3, E and F, and movie S3). Under tensile loading, the simulated force-displacement response exhibits five successive peaks corresponding to the snap opening of unit cells, one row at a time (Fig. 3E). The peak forces (7 to 8 mN) closely match experimental values (8 to 12 mN), confirming that the coupled elastic-magnetic lattice model quantitatively captures the sequential snapping mechanism.

### Reprogramming random-to-ordered snapping sequence by interlayer magnetic interactions

**Fig. 4.**
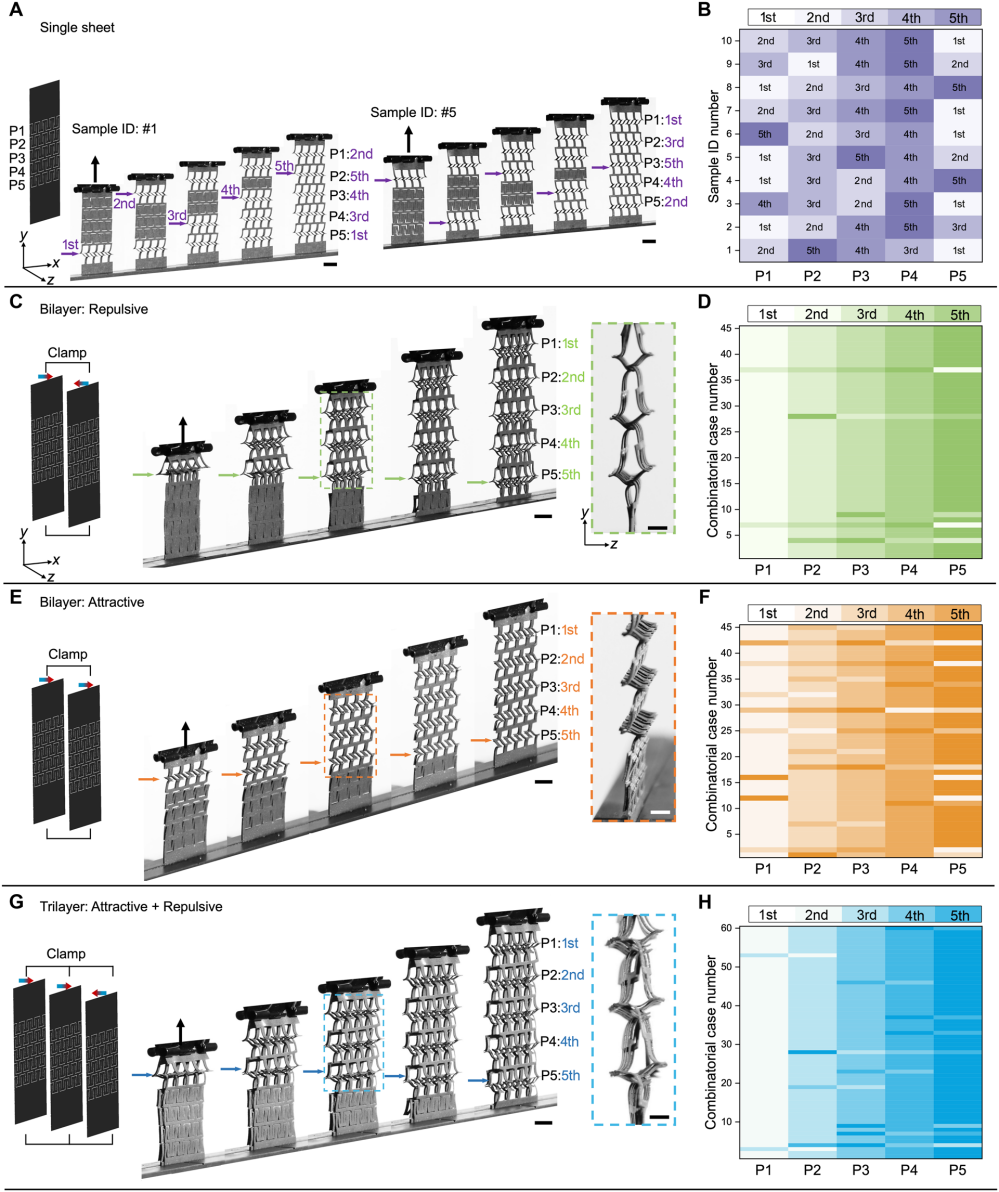
Random-to-ordered snapping in multilayer assemblies. (**A**) Snapshots of distinct snapping sequences in two representative single-layer magnetic samples (IDs 1 and 5), with unit-cell rows numbered P1 to P5 from top to bottom. (**B**) Snapping order contours across P1 to P5 rows for 10 independent single-layer samples. (**C** and **E**) Snapshots of ordered snapping propagating from top to bottom in repulsive (C) and attractive (E) bilayers with both ends clamped. (**D** and **F**) Corresponding snapping order contours for 45 combinatorial bilayers constructed from 10 single-layer samples. (**G**) Snapshots of ordered snapping in clamped trilayer assemblies. (**H**) Snapping order contours for 60 combinatorial trilayers from 10 single-layer samples.

Although single-layer metamaterials exhibit sequential snapping, the snapping order varies randomly between samples, as shown by the sequence contour plots of 10 tested samples (Fig. 4, A and B). Each row of pop-up struts is labeled from P1 to P5 (top to bottom), with the corresponding snapping order recorded (first to fifth). This is because experimental samples inevitably contain random geometric or material imperfections such as the nonuniform slit widths or gaps between struts as shown in Fig. 1C. The strong imperfection sensitivity of buckling instabilities in shells explains why randomness appears in experiments. Repeating the stretching cycles on the same sample produces identical snapping orders (fig. S7), confirming that randomness originates from fixed imperfections.

Strikingly, when individual single-layer samples are assembled into multilayer metamaterials with both ends clamped, ordered propagation emerges (Fig. 4, C to H). In bilayer structures, 45 combinatorial cases are possible for 10 independent samples ($C_{10}^{2}$), with interlayer interactions tuned to be either repulsive (Fig. 4, C and D) or attractive (Fig. 4, E and F) by flipping single layers. Trilayer structures have 60 possible combinations with mixed repulsive and attractive interactions (Fig. 4G). Despite random snapping sequences in single layers, bilayer structures display robust directional ordering: 40 of the 45 repulsive samples (88.9%; Fig. 4D) and 30 of the 45 attractive samples (66.7%; Fig. 4F) exhibit ordered snapping propagation. In trilayer structures, 48 (80%) of the 60 samples exhibit directional propagation (Fig. 4H). Interlayer magnetic interactions thus suppress imperfection-induced randomness and promote deterministic behavior.

**Fig. 5.**
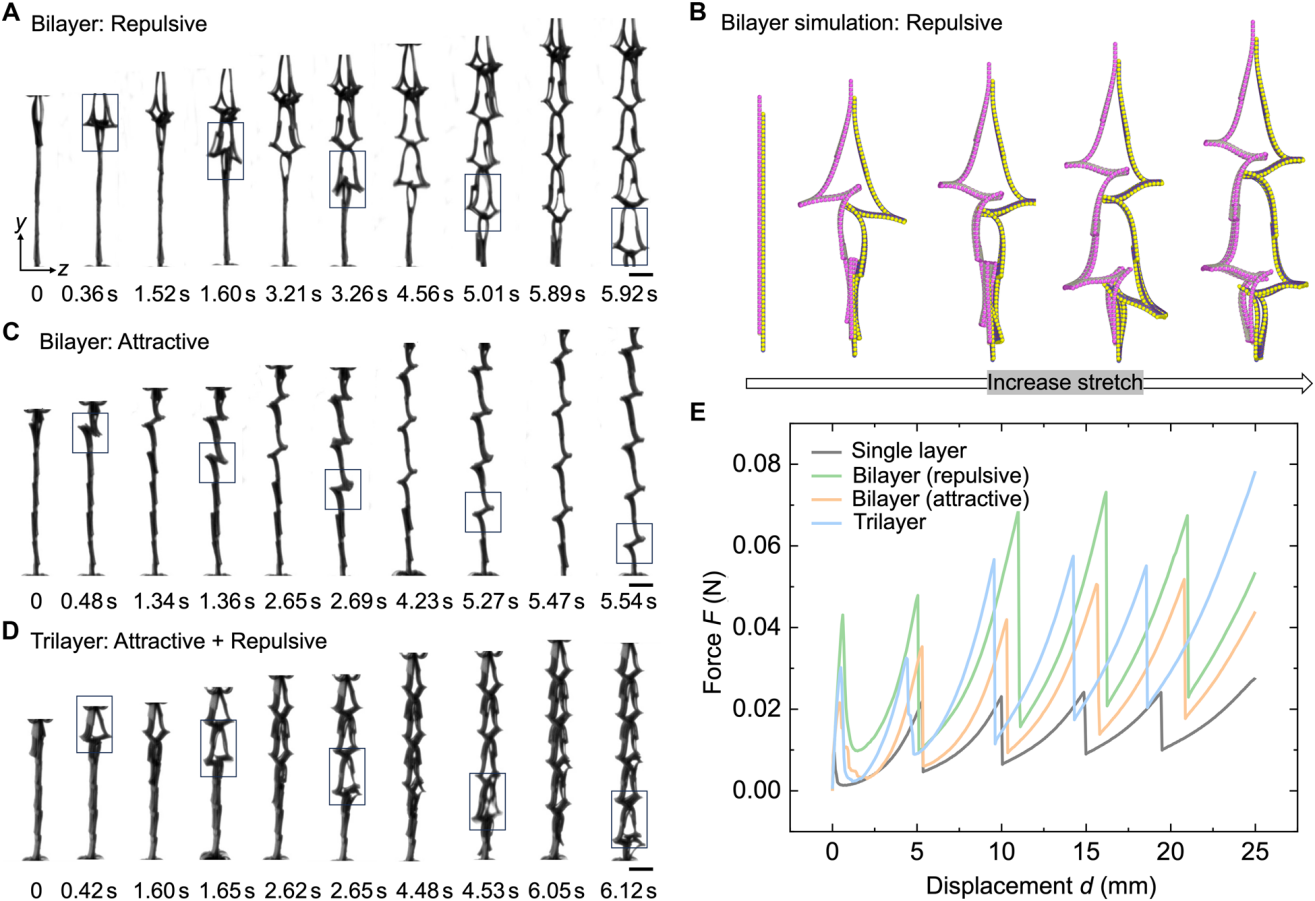
Deformation pathways and mechanical responses in multilayer assemblies. (**A**) Side-view time-lapse snapshots showing the directional chain reaction–like deformation pathway in a repulsive bilayer. (**B**) Simulated chain-reaction-like propagation in two-unit-cell repulsive bilayers. (**C** and **D**) Side-view time-lapse snapshots of directional pathways in an attractive bilayer (C) and in a trilayer combining repulsive and attractive layers (D). (**E**) Force-displacement curves comparing single-layer and multilayer assemblies. Scale bars, 5 mm [(A), (C), and (D)].

Among the three multilayer structures, repulsive bilayers exhibit the most robust ordering that originates from a chain reaction–like process (Fig. 5A and movie S4). Repulsive forces create an initial gap between the layers; with further stretching, this gap triggers neighboring struts to snap upward, forming a closed-bell-like opening in the adjacent row and initiating an additional gap underneath (Fig. 5A and right of Fig. 4C). This mechanism induces bending deformation across three rows simultaneously and is well reproduced in simulations (Fig. 5B and movie S5). As stretching continues, the chain reaction repeats row by row, driving directional propagation and enhancing ordering probability. The propagation is inherently directional due to the asymmetric bell shape set by the T-shaped cut pattern: Flipping the bilayer reverses the propagation direction, causing snapping to start from the opposite end (fig. S8 and movie S6).

By contrast, attractive bilayers do not exhibit chain reaction snapping (Fig. 5C and movie S7). Instead, both bonded layers in a single row snap together, forming an N-shaped profile without interlayer gaps (right of Fig. 4E). This geometry fails to trigger neighboring rows, resulting in lower ordering probability. Because of its symmetry, propagation always occurs from the stretching end toward the clamped end, regardless of sample orientation (fig. S8C). Magnetic attraction maintains close contact between the two layers throughout most of the stretching deformation (Fig. 5C), reducing the effective imperfection length scale from the slit width in single layers to the much smaller interlayer gap. This suppression of long-wavelength surface irregularities effectively filters out imperfections that would otherwise initiate alternative snapping pathways, enabling a preferred snapping route to emerge. As a result, the attractive configuration still exhibits a measurable degree of ordering (66.7%), albeit weaker than that observed in the repulsive case.

In trilayer structures, the ordering probability falls between that of purely attractive and purely repulsive bilayers, reflecting the interplay of opposing interactions. Chain reaction–like propagation is retained, with bell-shaped gaps emerging between the attractive bonded bilayer and the repulsive single layer, yielding robust and directional snapping sequences (Fig. 5D, fig. S8, right of Fig. 4H, and movie S8).

In addition to snapping order, interlayer interactions strongly influence the force-displacement (*F*-*d*) response compared to single-layer structures (Fig. 5E). While multilayer structures retain the same nonlinear staged *F*-*d* behavior as the single layer, their stiffness (slope) and critical buckling forces (spike height) do not increase monotonically with the number of layers. Instead, as the structure transitions from single layer to attractive bilayer and to trilayer, both stiffness and buckling force rise consistently. Notably, the repulsive bilayer exhibits the steepest slopes and highest spikes at each stage, indicating the largest energy barrier for initiating chain reaction–like propagation, a direct consequence of its repulsive interactions.

### Enhanced impact energy absorption through magnetic interactions

**Fig. 6.**
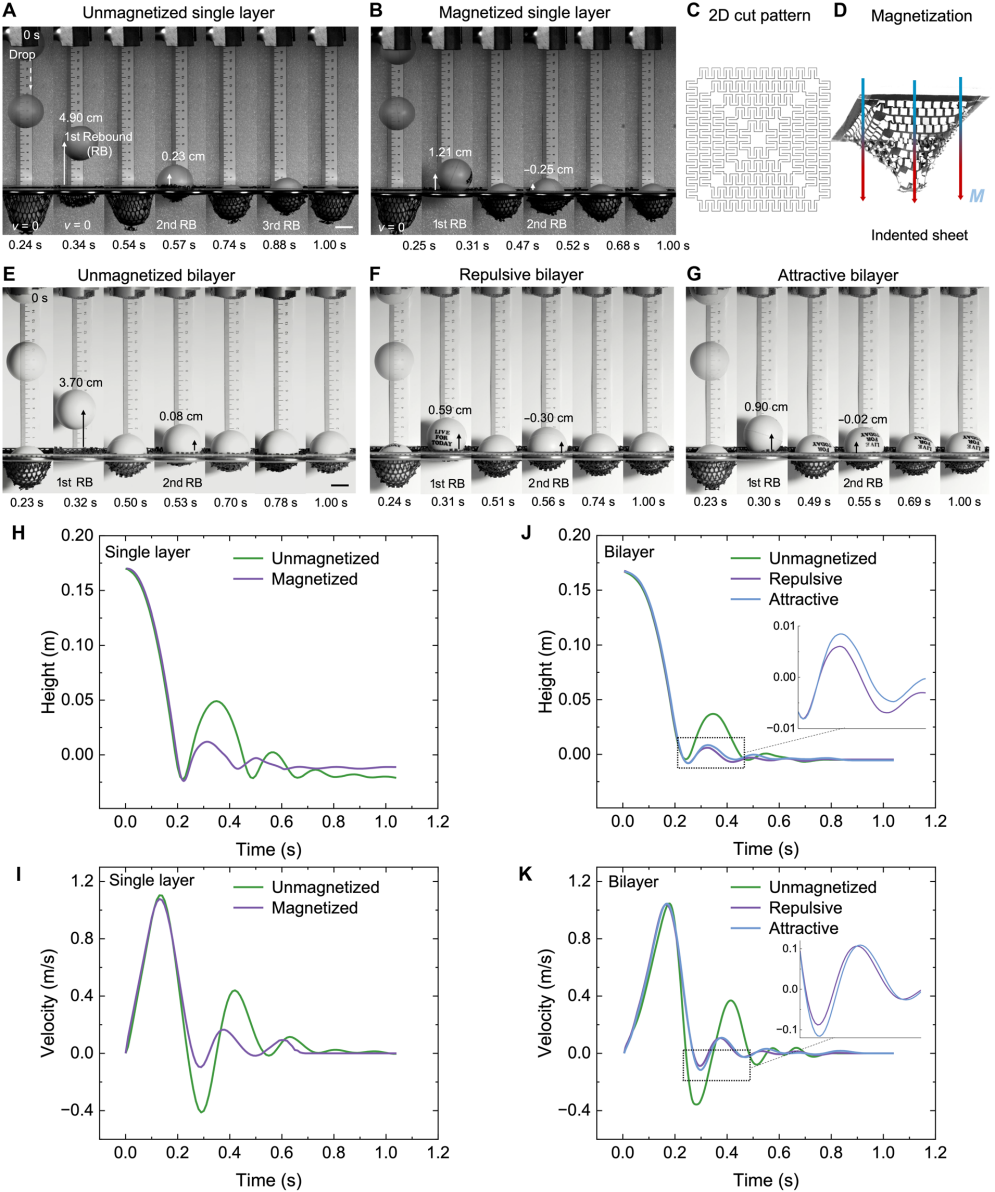
Enhanced energy absorption through internal magnetic interactions. (**A** and **B**) Drop test comparison between unmagnetized and magnetized 2D mesh-like single-layer metamaterials, with time-lapse snapshots at zero velocity during penetration and rebound. (**C** and **D**) Designed 2D cut pattern of the mesh (C) and schematics of magnetization of the sheet under indentation (D). (**E** to **G**) Drop test comparison among unmagnetized (E), magnetized repulsive (F), and attractive bilayer (G) metamaterials. (**H** and **I**) Time evolution of ball height (H) and velocity (I) during impact on unmagnetized and magnetized single-layer meshes. (**J** and **K**) Time evolution of ball height (J) and velocity (K) during impact on unmagnetized and magnetized bilayer meshes.

To harness the enhanced hysteresis from internal magnetic interactions, we investigate dynamic damping in a lightweight, mesh-like magnetic metamaterial (Fig. 6, A and B). The structure (2.26 g) is patterned with symmetric triangular T-cuts, fixed to a rigid frame, and designed to undergo biaxial stretching under impact (Fig. 6C). The sheet is magnetized under indentation in a stretched 3D pyramid shape (Fig. 6D and see Materials and Methods). A 7.5-g ball is released from a height of *H*_o_ = 0.17 m (impact speed of ~1.1 m/s), and the deformation dynamics is captured by high-speed imaging (Materials and Methods and movies S9 and S10). We compare two cases: an unmagnetized mesh and a magnetized mesh in the structures of a single layer (Fig. 6, A and B) and bilayer (Fig. 6, E to G).

Magnetic interaction markedly improves impact energy absorption. For a single layer, upon contact, the mesh deforms and wraps around the ball, dissipating energy through bending and stretching. During the first impact and rebound, the unmagnetized mesh exhibits deeper penetration (3.5 cm) and higher rebound (*H*_1−unmag_ = 4.9 cm) (Fig. 6, A and H), indicating an approximate energy absorption of *E*_abs−unmag_ ≈ mg(*H*_o_ − *H*_1−unmag_) = 8.9 mJ, consistent with its greater compliance and lower hysteresis. By contrast, the magnetized mesh halts the ball at shallower depths (3.0 cm) and suppresses the first rebound to near-negligible levels (*H*_1−mag_ = 1.2 cm) with reduced rebound velocities (Fig. 6, B, H, and I), indicating an approximate energy absorption of *E*_abs−mag_ ≈ mg(*H*_o_ − *H*_1−mag_) = 11.6 mJ. Thus, energy absorption increases by ~30% relative to the unmagnetized case during the first impact and rebound, reflecting the strong energy dissipation from magnetic coupling. Subsequent oscillations decay exponentially, and the magnetized mesh displays markedly diminished amplitudes (Fig. 6, H and I, and movie S9), confirming that internal magnetic interactions amplify hysteresis and impart enhanced damping capacity.

Similar enhancements in impact energy absorption resulting from internal magnetic interactions are also observed in multilayer structures (Fig. 6, E to G, and movie S10). The bilayer meshes exhibit much lower oscillation amplitudes and faster damping compared with the single-layer counterparts (Fig. 6, J and K). During the first cycle of impact and rebound, the magnetized repulsive and attractive bilayer meshes dissipate ~23.5% (12.1 mJ) and 20.4% (11.8 mJ) more energy, respectively, than the unmagnetized bilayer (~9.8 mJ) and roughly 35.6% more energy than the unmagnetized single layer (Fig. 6J and fig. S9).

Although the repulsive bilayer exhibits much higher critical buckling forces during quasi-static uniaxial stretching (Fig. 5E) for potentially greater energy dissipation, it shows only a marginal increase in energy absorption compared with the attractive bilayer and the magnetized single layer under dynamic impact loading. This discrepancy highlights the fundamentally different deformation mechanisms governing quasi-static and impact responses in multilayer magnetic meshes. Under quasi-static uniaxial loading, intra- and interlayer magnetic interactions promote programmed snapping sequences and well-defined deformation pathways. In contrast, during impact, these ordered sequences are effectively overridden by transient contact, penetration, and stress propagation initiated at the point of impact. The resulting strut opening is dominated by biaxial stretching of the mesh and by rapidly propagating local contacts, which proceed similarly in repulsive and attractive bilayers as well as in the single-layer mesh. Consequently, the programmed snapping order does not directly translate into enhanced impact resistance. Instead, impact resistance and energy absorption are governed primarily by intralayer magnetic interactions that resist strut opening within each layer. In the present samples, these interactions are relatively weak due to limitations in achieving sufficiently high magnetization intensity at large scales, which in turn constrains the overall energy absorption performance.

## DISCUSSION

We have demonstrated how internal magnetic interactions can drive random-to-ordered transitions in snapping and shape morphing within multilayer soft magnetic metamaterials. This ordering emerges from the interplay between elasticity and intra- and interlayer magnetic couplings, which establish directional, chain-reaction-like deformation pathways. We note that when magnetic coupling is weakened or when predefined geometric imperfections dominate, the deterministic ordering probability decreases remarkedly (Supplementary Materials). For example, reducing the original magnetization intensity *M* from 1.5 to 0.75 T lowers the ordering probability in repulsive bilayers from 88.9 to 22.2% (fig. S10A), as the diminished magnetic interactions make the chain reaction–like snapping process far less robust. Likewise, introducing a wider cut slit to the sheet as a large-amplitude geometric imperfection suppresses ordering, reducing the probability to 13.3% in repulsive bilayers (fig. S10B), where the imposed imperfection dominates the location of the first snap. Furthermore, when internal magnetic interactions are coupled with an externally applied magnetic field, the strong external field dominates, overriding the internal interactions. Consequently, sequential snapping is suppressed and replaced by a simultaneous buckling mode with enhanced hysteresis (fig. S11 and movie S11). The combined coupling with the external field also yields a modest increase in energy absorption (fig. S12 and movie S9).

Extending this principle offers a route to stable and multistable, fully soft, reconfigurable 3D magnetic architectures (*43*) with enhanced stiffness for loadbearing capabilities and deterministic responses, all without the need for a continuously applied external field. By coupling mechanics and magnetism in this manner, our approach lays the groundwork for architected materials with broad potential, spanning dynamic waveguiding, energy dissipation, reconfigurable soft robotics, mechanical computing, and biomedical devices.

## MATERIALS AND METHODS

### Fabrication of planar magnetic metamaterials

The base elastomeric composite was prepared by blending uncured Dragon Skin 30 (Smooth-On Inc., part A and part B mixed at a 1:1 weight ratio) with neodymium-iron-boron (NdFeB) magnetic microparticles (MQFP-B + 20174-088, Magnequench, average particle diameter of 25 μm) at a loading ratio of 60 weight % relative to the elastomer. The mixture was thoroughly mixed and then degassed under vacuum for 5 min to remove entrapped air. A total of 5.0 g of the composite was dispensed into a petri dish (100 mm by 50 mm) and spin-coated using a spin coater (EZ4, Schwan Technology) at 1000 rpm for 20 s. The coated films were cured on a hot plate (Guardian 5000) at 55°C for 1 hour. After curing, the sheets were cut into prescribed patterns using a CO_2_ laser cutter (40 W; Epilog Laser).

### Magnetization process

The unmagnetized sheet was mechanically prestretched to generate a prescribed tilting angle, and then magnetized between the poles of a GMW 3472-70 electromagnet. Magnetization was performed under a uniform magnetic field ranging from 0.5 to 1.75 T.

To magnetize the 2D kirigami sheet, a 3D printed indenter (length = 4.5 cm) was positioned at the center of the sheet. The boundary of the sheet was fixed to the bottom plate of the indenter to maintain the kirigami pattern in an opened and stretched state. The resulting pyramidal structure was then placed into the electromagnet, with the magnetic field direction aligned along the symmetry axis of the geometry. Magnetization was conducted under a field strength of ~0.94 T.

### Magnetic field imaging

Magnetic fields of the samples were imaged using a CMOS-MagView S magnetic field camera equipped with a type-B magneto-optical sensor (20 mm by 15 mm), providing a spatial resolution of 25 μm and having a saturation field of 65 mT. The device uses the Faraday effect to visualize magnetic fields and, following an appropriate calibration, allows the determination of the magnetic field component perpendicular to the sensor surface. This setup enabled high-resolution mapping of the magnetic structures under investigation.

### Force-displacement measurement

The mechanical properties of magnetic metamaterials were characterized using uniaxial tensile tests conducted on an Instron 5944 machine equipped with a 5-N load cell. The sample boundaries were clamped between custom fixtures to ensure uniform loading, and displacement-controlled loading was applied at a constant rate of 8 mm/min. To examine responses under a localized external magnetic field, a bar-shaped permanent magnet (MKD60103-6P, N45 grade; 60 mm by 10 mm by 3 mm) was positioned parallel to the major axis of metamaterial using a 3D printed holder. The holder enabled precise adjustment of the magnet-to-sample distance, which was varied between 3 and 7 mm during testing.

### High-speed motion capture and data analysis

The sequential snapping of the magnetic metamaterials was captured using a high-speed camera (Photron SA-2), with a frame rate ranging from 500 to 1000 fps. The position information was captured with Tracker.

### FEA simulation

Quasi-static FEA simulations were performed in Abaqus/Explicit to examine the mechanical response of the unmagnetized structures under uniaxial loading. The model used C3D8R solid elements, with the material defined as linear elastic and isotropic (Young’s modulus: 1.5 MPa; Poisson’s ratio: 0.48). The linear buckling analysis is conducted in Abaqus/Standard to extract the critical buckling modes and related critical buckling force/strain and introduced small imperfections to trigger buckling in the following quasi-static simulation. A tensile length of 20 mm was prescribed, and the displacement loading was applied along the designated axis. Boundary conditions were defined to replicate realistic constraints while permitting the onset and evolution of buckling and postbuckling behavior.
