# Supplementary material for: Magnetic coupling transforms random snapping into ordered sequences in soft metamaterials
Source: Sci Adv. 2026 Mar 20;12(12):eaec3182. doi: 10.1126/sciadv.aec3182 (PMC13004017; doi:10.1126/sciadv.aec3182)
Supplement: Supplementary file 1 — Supplementary Text Figs. S1 to S12 Legends for movies S1 to S11 [file sciadv.aec3182_sm.pdf]

Science Advances
AAAS

Supplementary Materials for

Magnetic coupling transforms random snapping into ordered sequences in soft metamaterials

Haoze Sun *et al.*

Corresponding author: Jie Yin, jyin8@ncsu.edu

Sci. Adv. **12**, eaec3182 (2026)
DOI: 10.1126/sciadv.aec3182

The PDF file includes:

Supplementary Text
Figs. S1 to S12
Legends for movies S1 to S11

Other Supplementary Material for this manuscript includes the following:

Movies S1 to S11

Triangular lattice models

We develop a simulation model with springs, dihedral angles, and dipoles to capture the nonlinear and nonlocal interplay between elastic deformation and magnetic interactions (**Fig. 3A**). We implement the model in the LAMMPS software (*39*). The elastic model in the main text is only valid for a perfect triangular lattice, which is only possible for a sheet with hexagonal symmetry. For an irregular mesh, there are many ways to approximate the stretching and bending energies. We use the bending energy from (*38*) and a simplified stretching energy since the deformation is bending dominated (*35-38*)

$$U_E = \frac{\sqrt{3}}{4} Et \sum_{\text{edges}} (l_e - l_e^0)^2 + \frac{2}{\sqrt{3}} K \sum_{\text{dihedrals}} \frac{l_d^2}{A_d/3} (1 - \boldsymbol{n}_\alpha \cdot \boldsymbol{n}_\beta) \quad \text{(S1)}$$

where E is the Young modulus, $K = \frac{Et^3}{12(1-\nu^2)}$ is the bending modulus, ν is the Poisson ratio, t is the thickness, l_e^0 and l_e are the initial and current bond length of edge e, and $\boldsymbol{n}_\alpha$ and $\boldsymbol{n}_\beta$ are the normal of the dihedral d with edge length l_d and area A_d. The area is divided by 3 because each interior triangle is shared by 3 dihedrals.

We introduce a physical dipole node at the center of each triangle to implement the non-local magnetic dipole interaction. To couple the magnetic dipole with the elastic deformation of the thin shell, we adopt the following method to constrain the dipole location and orientation, such that

$$U_P = \sum_\alpha \frac{1}{2} k_c \left\| \boldsymbol{x}_\alpha - \bar{\boldsymbol{x}}_\alpha^{ijk} \right\|^2 + \sum_\alpha \frac{1}{2} k_d \left[\left(\phi_\alpha^i - \phi_\alpha^{i\,0} \right)^2 + \left(\phi_\alpha^j - \phi_\alpha^{j\,0} \right)^2 + \left(\phi_\alpha^k - \phi_\alpha^{k\,0} \right)^2 \right], \quad \text{(S2)}$$

where $\boldsymbol{x}_\alpha$ is the dipole position, $\bar{\boldsymbol{x}}_\alpha^{ijk}$ is the centroid of the triangle Δ_α^{ijk}, k_c is a penalty stiffness to maintain the dipole node at the center of the triangle, ϕ_α^i and $\phi_\alpha^{i\,0}$ are the current and initial angle between the dipole moment vector $\boldsymbol{m}_\alpha$ and the vector $\boldsymbol{r}_{i\alpha} = \boldsymbol{r}_i - \boldsymbol{r}_\alpha$, and k_d is a penalty stiffness to transfer the torque between the dipole and the mechanical node.

The physical dipole nodes have a finite radius to incorporate torques. The dipole moment of each dipole node is given by $\boldsymbol{m} = \boldsymbol{M}At$, where $\boldsymbol{M}$ is the magnetization, A is the area, and t is the thickness of the corresponding element, all assumed to be constant within the element. Magnetic dipoles interact according to the well-known dipole-dipole energy (*42*)

$$U_M = \sum_{\alpha \neq \beta} \frac{\mu_0}{4\pi r_{\alpha\beta}^3} \left[\boldsymbol{m}_\alpha \cdot \boldsymbol{m}_\beta - 3(\boldsymbol{m}_\alpha \cdot \hat{\boldsymbol{r}}_{\alpha\beta})(\boldsymbol{m}_\beta \cdot \hat{\boldsymbol{r}}_{\alpha\beta}) \right], \tag{S3}$$

where μ_0 is the vacuum magnetic permeability, $\boldsymbol{r}_{\alpha\beta} = \boldsymbol{r}_\alpha - \boldsymbol{r}_\beta$ and $\hat{\boldsymbol{r}}_{\alpha\beta} = \frac{\boldsymbol{r}_{\alpha\beta}}{r_{\alpha\beta}}$.

The force and torque on particle α due to particle β are

$$\boldsymbol{F}_{\alpha\beta}^M = \frac{3\mu_0}{4\pi r_{\alpha\beta}^4} \left[(\boldsymbol{m}_\alpha \cdot \boldsymbol{m}_\beta)\hat{\boldsymbol{r}}_{\alpha\beta} + (\boldsymbol{m}_\alpha \cdot \hat{\boldsymbol{r}}_{\alpha\beta})\boldsymbol{m}_\beta + (\boldsymbol{m}_\beta \cdot \hat{\boldsymbol{r}}_{\alpha\beta})\boldsymbol{m}_\alpha - 5(\boldsymbol{m}_\alpha \cdot \hat{\boldsymbol{r}}_{\alpha\beta})(\boldsymbol{m}_\beta \cdot \hat{\boldsymbol{r}}_{\alpha\beta})\hat{\boldsymbol{r}}_{\alpha\beta} \right], \tag{S4}$$

$$\boldsymbol{T}_{\alpha\beta}^M = \frac{\mu_0}{4\pi r_{\alpha\beta}^3} \left[3(\boldsymbol{m}_\beta \cdot \hat{\boldsymbol{r}}_{\alpha\beta})(\boldsymbol{m}_\alpha \times \hat{\boldsymbol{r}}_{\alpha\beta}) - (\boldsymbol{m}_\alpha \times \boldsymbol{m}_\beta) \right]. \tag{S5}$$

The computation of this energy is expensive due to its long-ranged nature, so we only consider interactions within a finite cutoff. We chose a cutoff r_{cut} as 10 times the average distance between neighboring dipoles, which provides a good compromise between speed and accuracy. We also exclude the first nearest neighbor interactions to make the simulations stable.

To gain insight into the chain-reaction like kirigami opening in the repulsive bilayer, we use short krigami sheets with two cuts that can reproduce the opening behavior with minimal computational cost. It is found that adhesive interaction should be included in the bilayer structure to maintain a stable initial configuration, which is modeled using Lennard-Jones potential. The separation of the two sheets is set to be the film thickness as 0.25 mm.

Simulation parameters

For the simulation in Fig. 3D-3E, a Young's modulus $E = 1.5$ MPa and thickness $t = 0.25$ mm

were used. The penalty parameters were set to $k_c = 300$ N/m, approximately twice the stretching stiffness ($\sim \frac{\sqrt{3}}{4} Et$), and $k_d = 40K$, where $K = \frac{Et^3}{12(1-\nu^2)}$ is the bending stiffness. The remanent magnetic field was set to $B^r = 106$ mT, corresponding to a magnetization of $M = B^r/\mu_0 = 84.35$ kA/m.

The kirigami sheet was quasi-statically deformed at a rate of 1 mm/s by prescribing equal and opposite constant velocities at the two ends. The unconstrained portion was advanced using explicit time integration, with forces and torques computed from Eqs. S1–S5. A global Langevin damping term was included to dissipate high-frequency oscillations, and gravity was applied as a uniform body acceleration. Fig. 3D shows the snapshots at different displacements. We note that the current model becomes unstable at higher magnetization, potentially limiting the parameter space for further reducing discrepancies between simulations and experiments. Future studies will focus on improving model stability.

Magnetization patterns

We follow the same magnetization process in experiments to create the initial magnetic profile. The non-magnetized planar metamaterial is initially stretched to 90° and the rotation of each element is calculated. In the stretched configuration, we assume that each dipole moment will be aligned with the external magnetic field. The orientation of the dipoles in the reference configuration is then approximately obtained by applying the inverse rotation to the orientation of the magnetizing field.

Assuming that in this stretched configuration the dipoles are aligned with the magnetizing field, the orientation of the dipoles in the reference configuration is simply the inverse rotation applied to the current magnetizing direction. For example, Fig. S5 shows the magnetization process for

the sheet with a 90° tilting angle. The dipole distribution is also visualized in Fig. 3(A) (top view) and Fig. S5 (side view) by extracting the out-of-plane magnetic field component (B_z) near the surface. Here, we construct a 3D finite magnetic film from the triangular lattice network by extruding the surface along its normal direction with a thickness of 0.25 mm and then discretizing it into a tetrahedral mesh. Using the 3D mesh structure, we compute the magnetic fields using Magpylib [6]. Fig. 3C shows simulated surface B_z using the 3D model at a distance $d = 0.15$ mm above the surface.

Effects of magnetic strength and geometric imperfections on the ordering probability

Two sets of statistical experiments were conducted by lowering the interlayer magnetic strength (Fig. S10A) and purposefully enlarging imperfection amplitudes (Fig. S10B-C) to explore their effects on the ordering probability.

First, to probe the role of magnetic coupling strength, we reduced the magnetization field from 1.5 T to 0.75 T and repeated the bilayer–repulsive statistical test. Lowering the magnetization significantly increased the randomness of the snapping order and reduced the robustness of the chain-reaction-like snapping pathways, reducing the ordering probability to 22.2% (Fig. S10A). Gauss-meter measurements of the remanent surface flux density (Fig. S10D) confirm that 0.75 T magnetization yields substantially weaker remnant magnetization than fields ≥1 T.

Second, to evaluate the influence of geometric imperfections, we introduced a controlled defect by manually enlarging the cut width of the third row to 350 μm (compared with the ~80 μm laser-cut gap) in ten bilayer–repulsive samples (Fig. S10B-C). As shown in Fig. S10B, this localized imperfection strongly biases the snapping sequence: in 45 independent trials, the first snapping event occurred predominantly at the defect row, and the overall ordering probability (defined as

propagation from row 1 to row 5 without disruption) decreased to 13.3%.

Together, these experiments provide direct quantitative evidence that ordered snapping is jointly regulated by magnetic strength and imperfection amplitude: stronger magnetic coupling suppresses defect-driven randomness, whereas larger imperfections or insufficient magnetization undermine the geometric stability required for deterministic propagation.

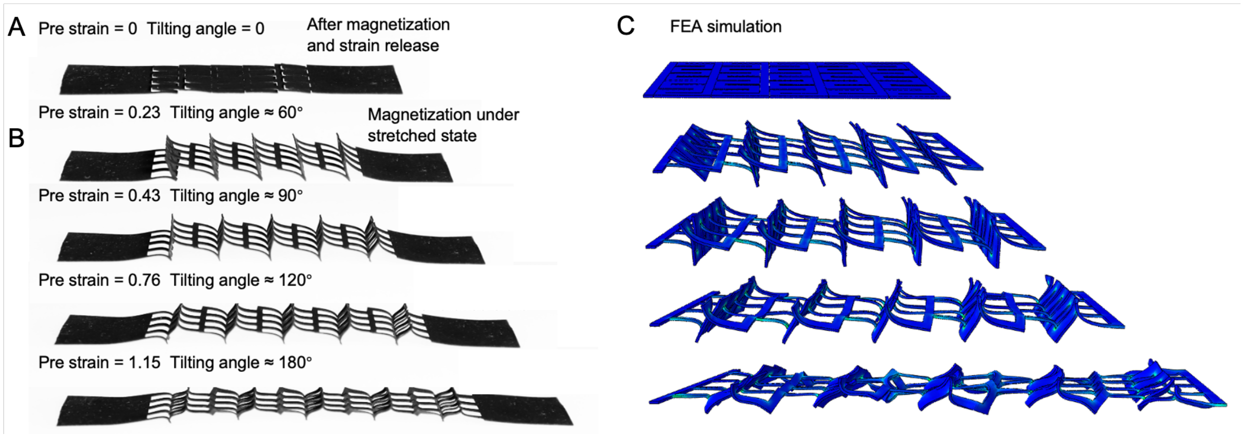

Fig. S1. Geometry evolution of the sample under various prestrain levels. (A) The zero-pre-strain magnetized sample shows repulsive magnetic interactions. (B) Experimental images showing the magnetization states under varying pre-strains with increasing tilting angles. (C) The corresponding finite element analysis (FEA) simulation results on the tilting angle changes under the same pre-strain conditions as in the experiments.

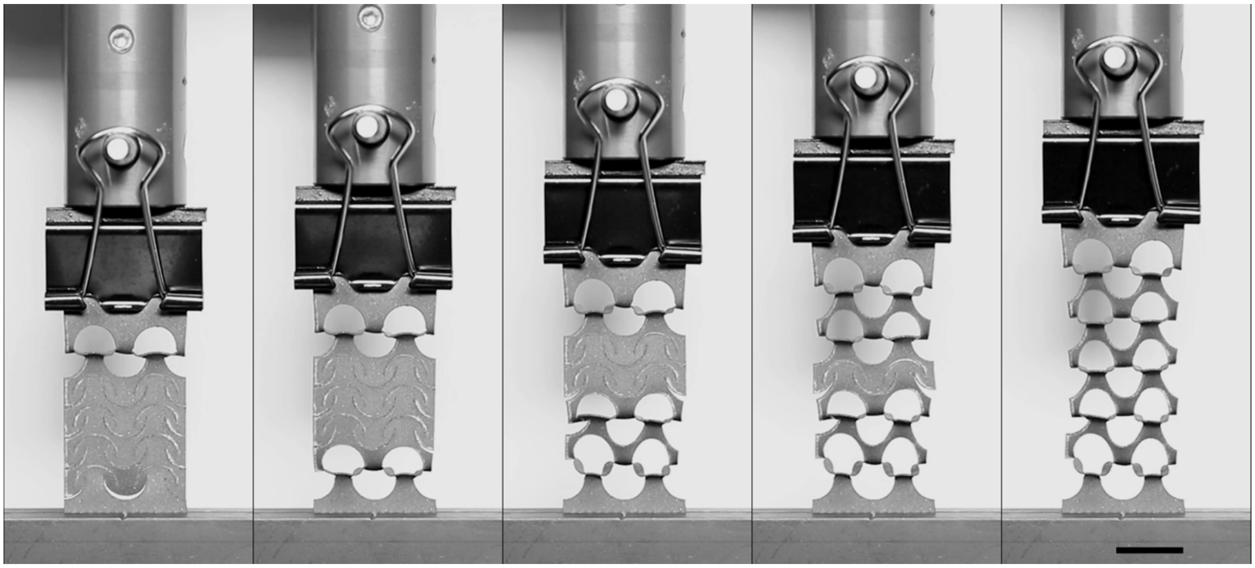

Fig. S2. Sequential snapping of a curved cut motifs during uniaxial tension. Scale bar, 10 mm.

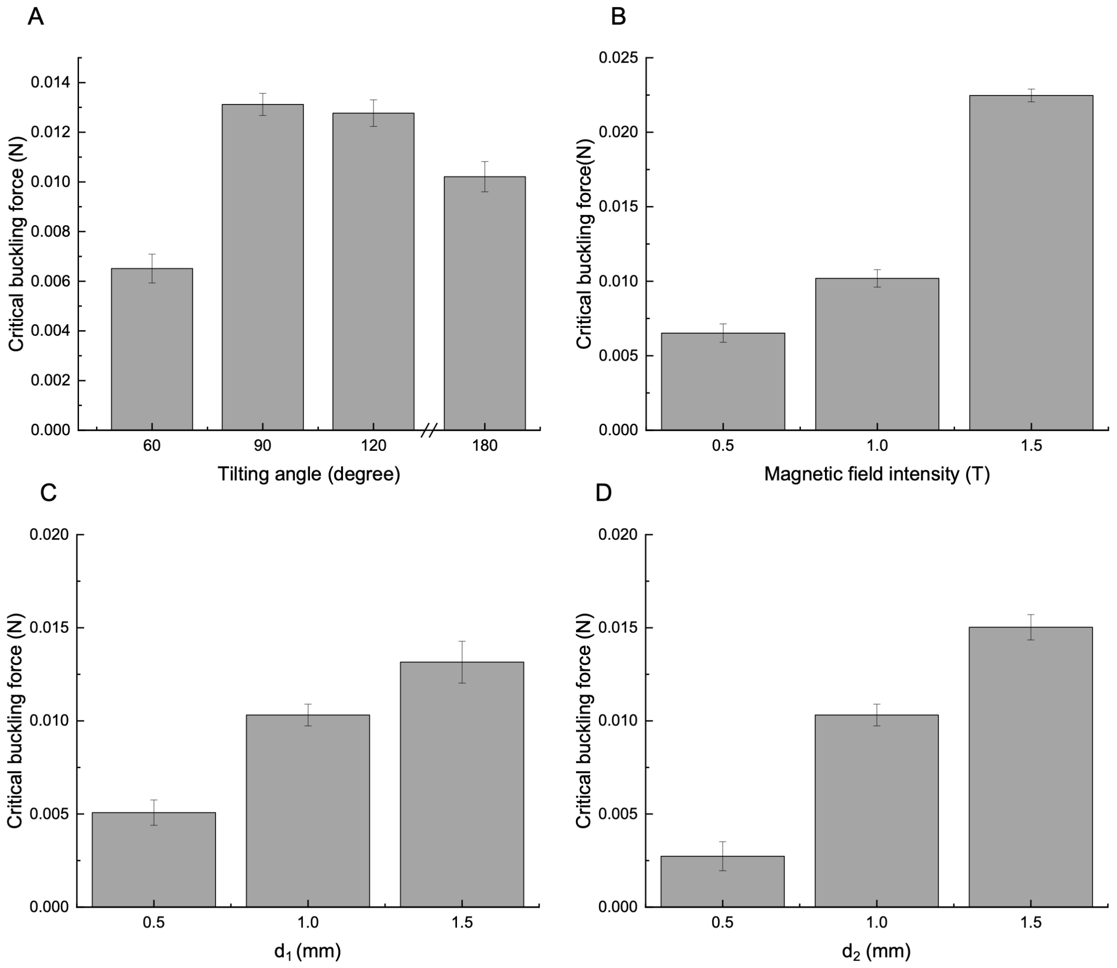

Fig. S3. Comparison of critical buckling force under various parameters. (A) Influence of tilting angle (pre-strain level) during magnetization on the critical buckling force. (B) Influence of magnetic field intensity during magnetization on the critical buckling force. (C) Changes in critical buckling force with variations in junction width d_1. (D) Changes in critical buckling force with variations in ribbon width d_2.

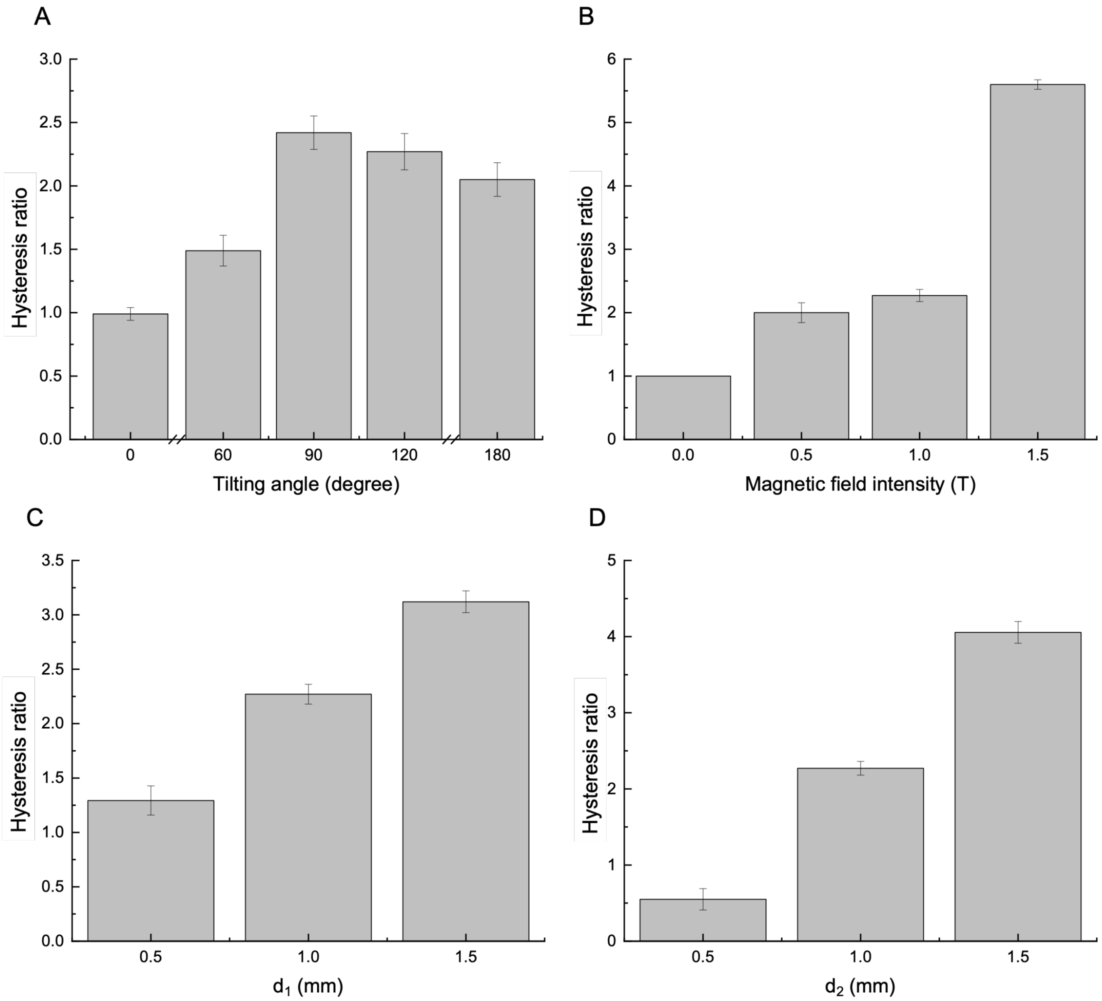

Fig. S4. Comparison of hysteresis area ratios under various parameters. (A) Effect of different tilting angles (pre-strain levels) during the magnetization process on the hysteresis area ratio. (B) Impact of different magnetic field intensities during the magnetization process on the hysteresis area ratio. (C) Variation in hysteresis area ratio with changes in junction width d_1. (D) Variation in hysteresis area ratio with changes in ribbon width d_2.

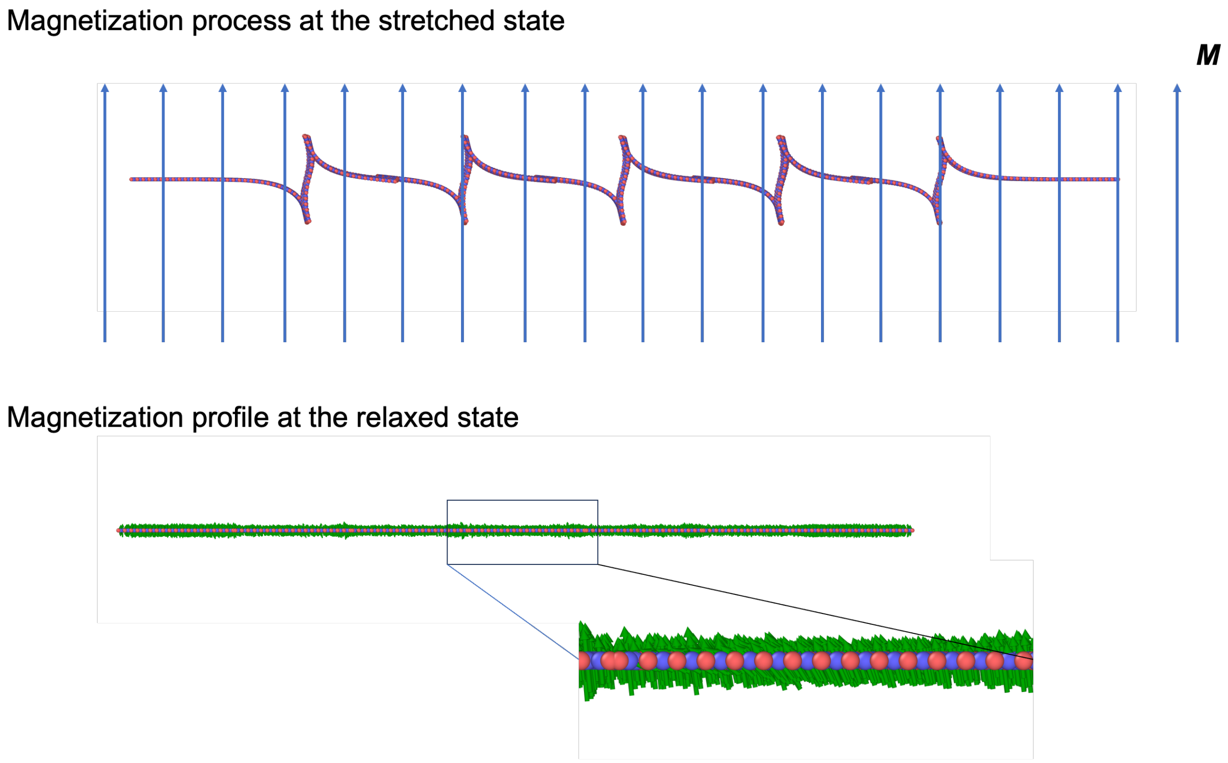

Fig. S5. Schematic of the magnetization process in simulation. Magnetization at the stretched state (top) and magnetic dipoles at the reference state (bottom).

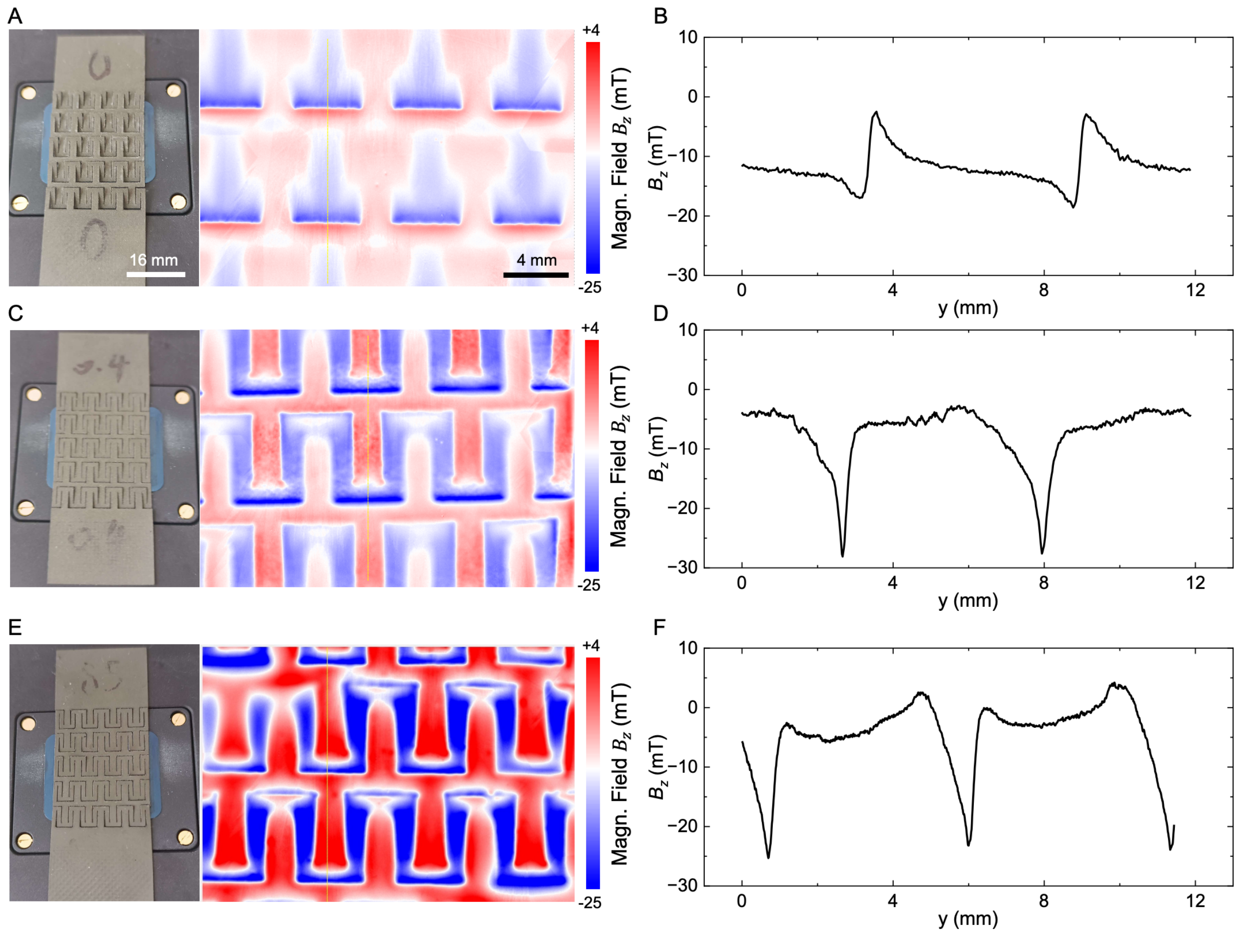

Fig. S6. Magnetic-field mapping of magnetized kirigami sheets under varying pre-strains. (A) Optical image of the magnetized kirigami sheet at 0 pre-strain (scale bar: 16 mm) placed on the detector (blue window) of the magnetic-field viewer, along with the experimentally measured spatial distribution of the out-of-plane magnetic field component (B_z). Scale bar: 4 mm. (B) Corresponding magnetic-field profile extracted along the vertical yellow line in panel (A). (C) Optical image of the magnetized kirigami sheet at 0.4 pre-strain with its measured B_z field map. (D) Field profile taken along the yellow line indicated in panel (C). (E) Optical image of the magnetized kirigami sheet at 0.85 pre-strain with its measured B_z distribution. (F) Field profile extracted along the yellow line shown in panel (E).

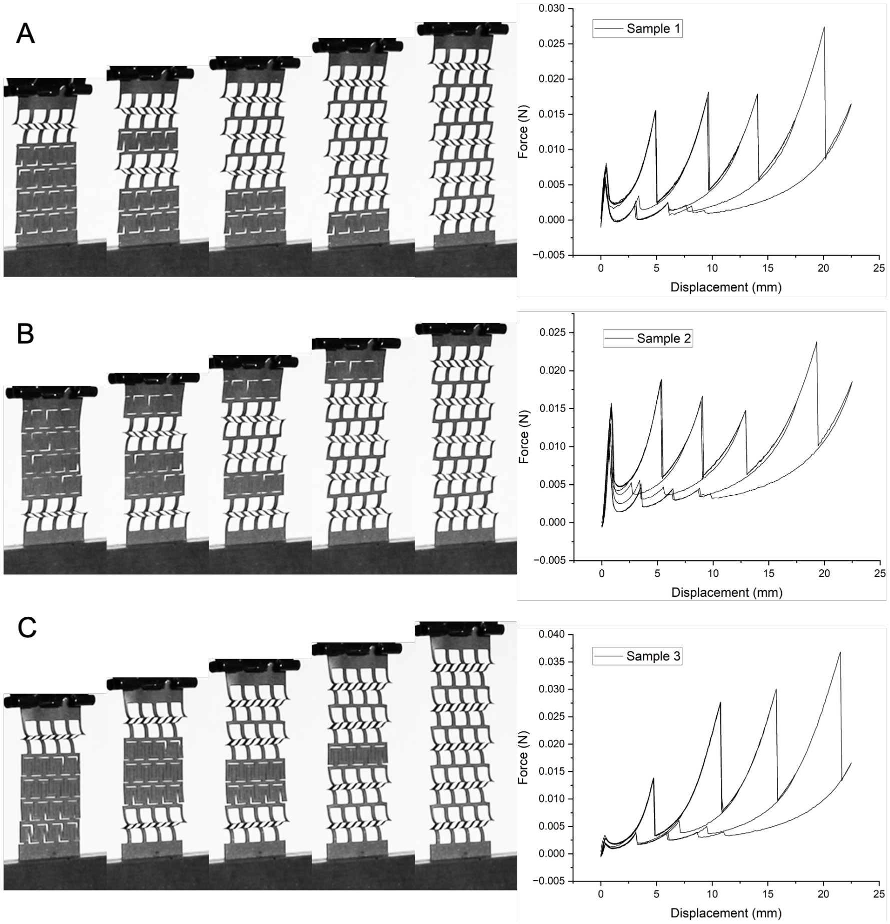

Sample 1	1st	2nd	3rd	4th	5th
Test 1	P1	P3	P2	P4	P5
Test 2	P1	P3	P2	P4	P5
Test 3	P1	P3	P2	P4	P5
Test 4	P1	P3	P2	P4	P5
Test 5	P1	P3	P2	P4	P5

Sample 2	1st	2nd	3rd	4th	5th
Test 1	P5	P2	P3	P4	P1
Test 2	P5	P2	P3	P4	P1
Test 3	P5	P2	P3	P4	P1
Test 4	P5	P2	P3	P4	P1
Test 5	P5	P2	P3	P4	P1

Sample 3	1st	2nd	3rd	4th	5th
Test 1	P1	P5	P2	P4	P3
Test 2	P1	P5	P2	P4	P3
Test 3	P1	P5	P2	P4	P3
Test 4	P1	P5	P2	P4	P3
Test 5	P1	P5	P2	P4	P3

Fig. S7. Sequential response of single-sheet magnetic metamaterial in uniaxial tension tests. (A–C) Snapshots from experimental sequences and corresponding force–displacement curves for three different samples. Results from five repeated tests for each sample are included to demonstrate consistency in sequential behavior on the right.

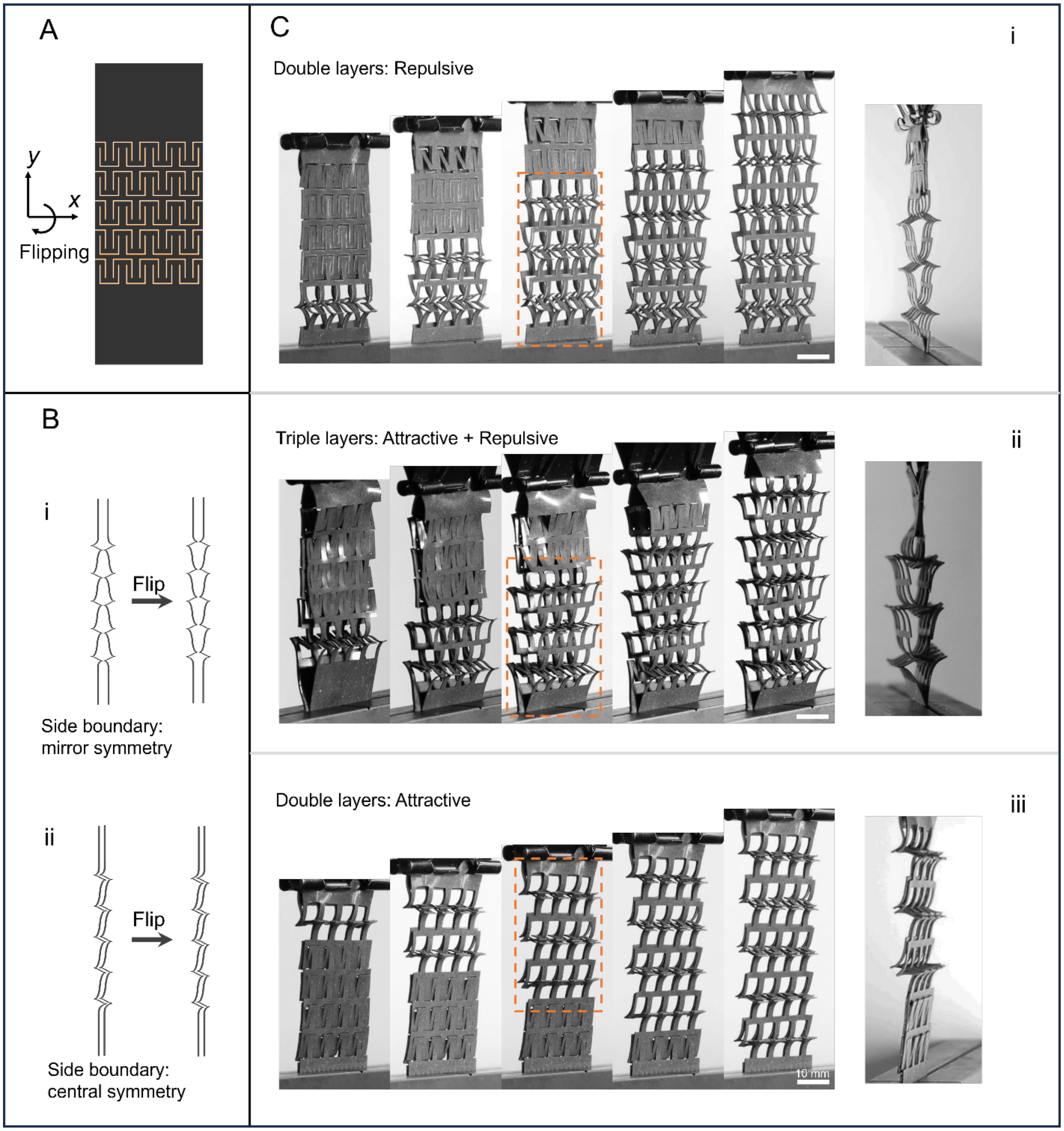

Fig. S8. Influence of flipping orientation on sequential snapping in multilayer samples. (A) Front view schematics showing the flipping directions of the metamaterial. (B) Side view schematics showing the flipping directions of the bilayer metamaterial. (i) Side boundary of repulsive bilayer structure. (ii) Side boundary of attractive bilayer structure. (C) Side-view illustrations showing the effect of top-to-bottom flipping on sequential activation in the three multi layer structures. (i) Sequential order changes in the bilayer repulsive case. (ii) Sequential order changes in the trilayer case. (iii) Sequential order remains unchanged upon flipping in the bilayer attractive configuration.

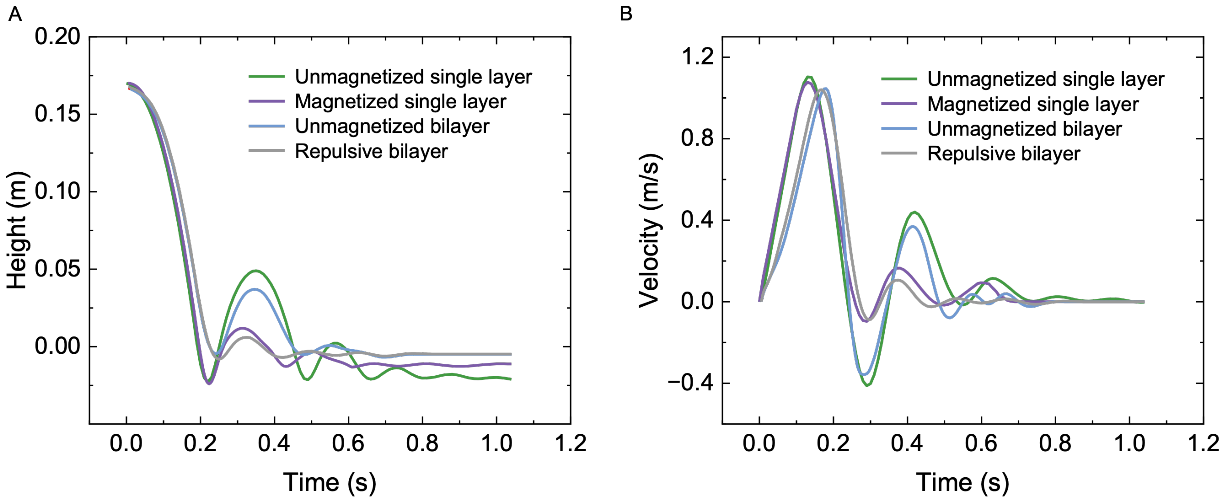

Fig. S9. Comparison of height and velocity during impact tests of unmagnetized and magnetized single and bilayers. (A-B) Time evolution of ball height (A) and velocity (B) during impact.

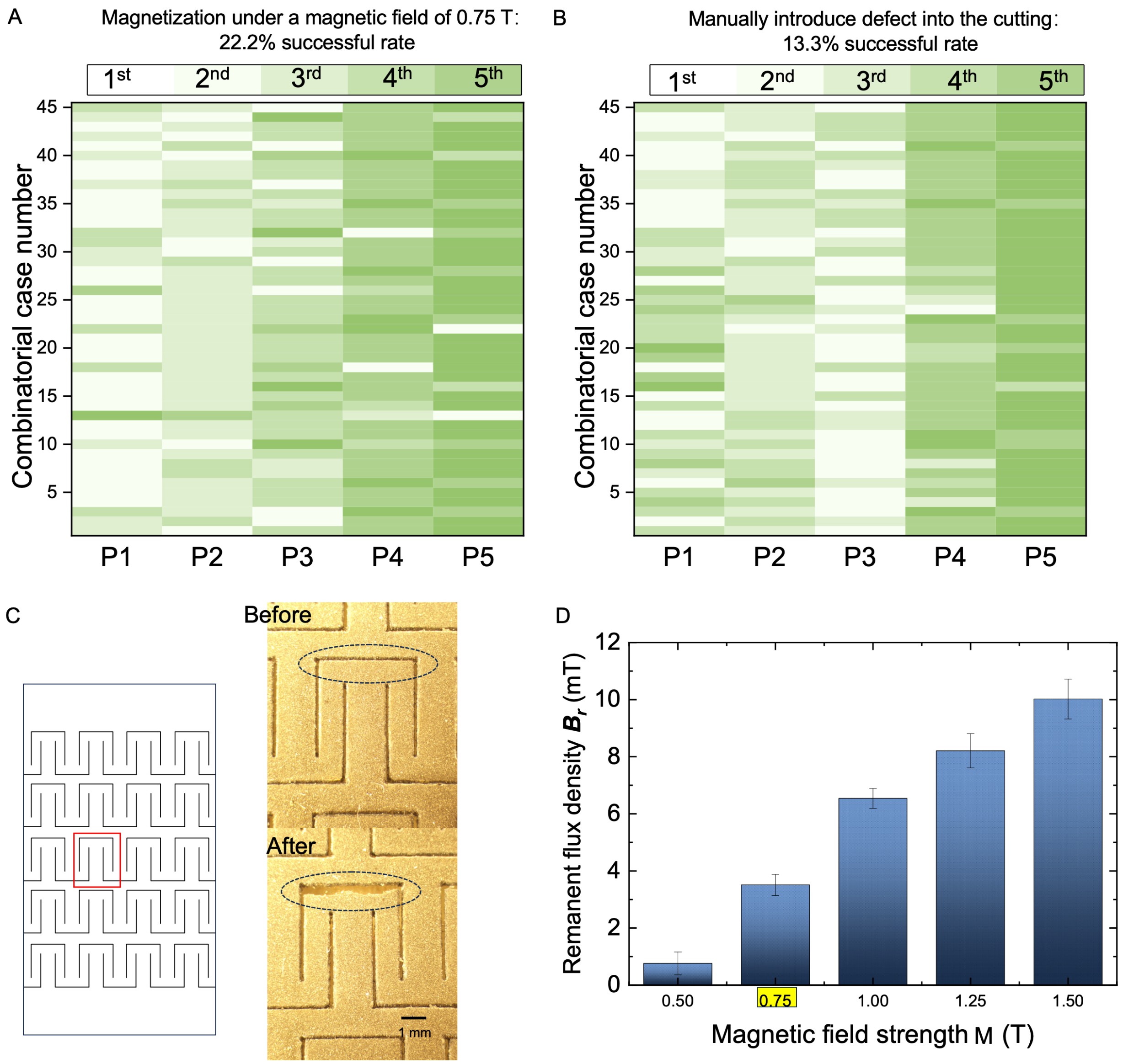

Fig. S10. Effects of magnetic strength and geometric defects on the ordering probability. (A) Snapping order contours across P1–P5 rows for 10 independent single-layer samples magnetized under a relatively weaker field of 0.75 T. (B) Snapping order contours across P1–P5 rows for 10 independent single-layer defected samples. (C) The defected sample with an introduced wider slit of 350 μm at the third row. The normal slit width is about 80 μm. (D)The remanent flux density in the magnetized samples under varying magnetization field strengths.

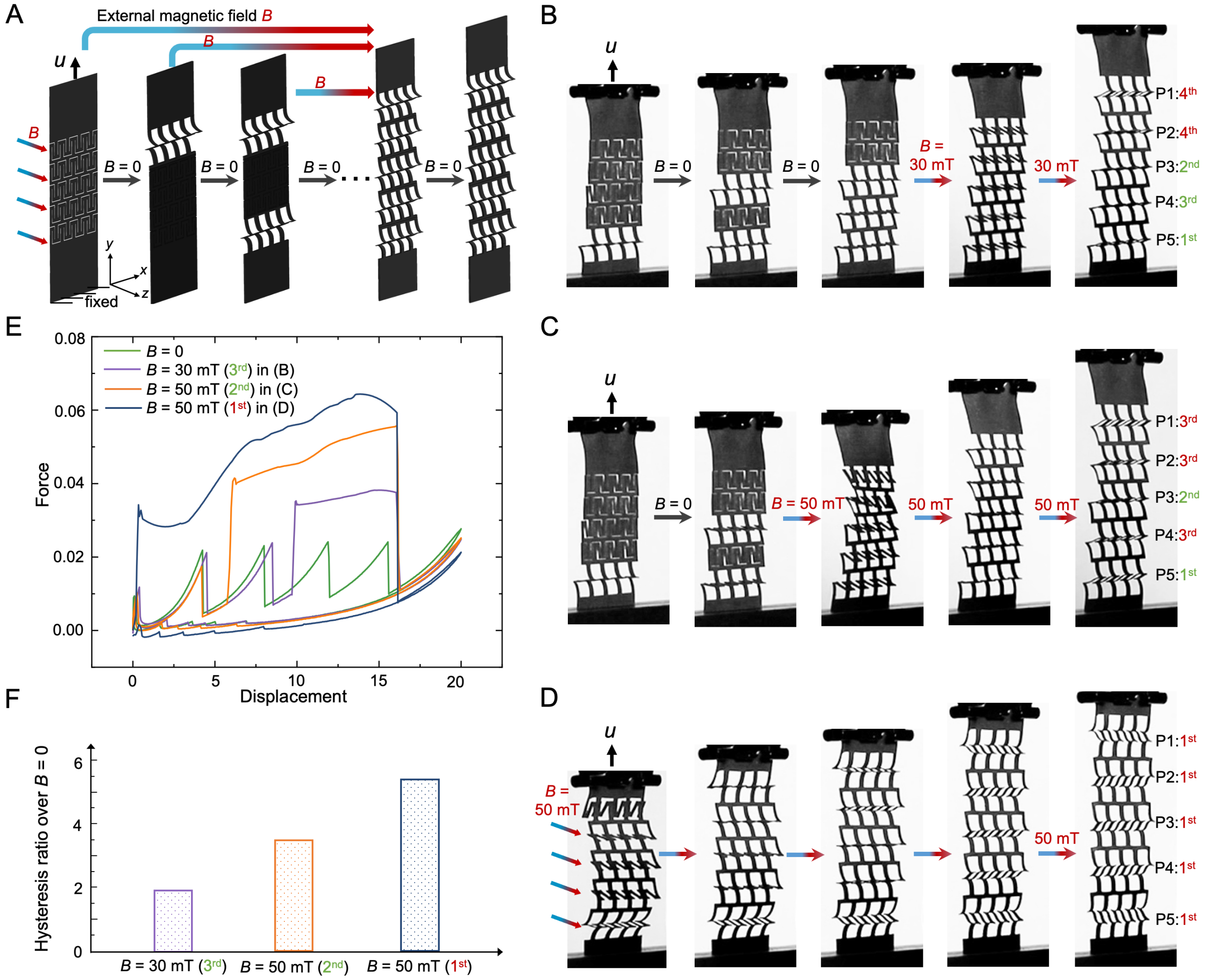

Fig. S11. Effects of local external magnetic fields on the sequential deformation and hysteresis of magnetic metamaterials. (A) Schematic illustration of the application of a localized external magnetic field and its influence on the snap-through sequence. (B-D) Experimental snapshots showing the sequential snap-through order is altered by the presence of localized magnetic field at different intervention time. (E) Stress–strain curves show that varying the intensity of the applied local magnetic field (as illustrated in B–D) modifies the hysteresis behavior, compared with that of a metamaterial magnetized at 1.75 T without an external field. (F) Comparison of hysteresis ratio over $B = 0$ under three different magnetic field intensities.

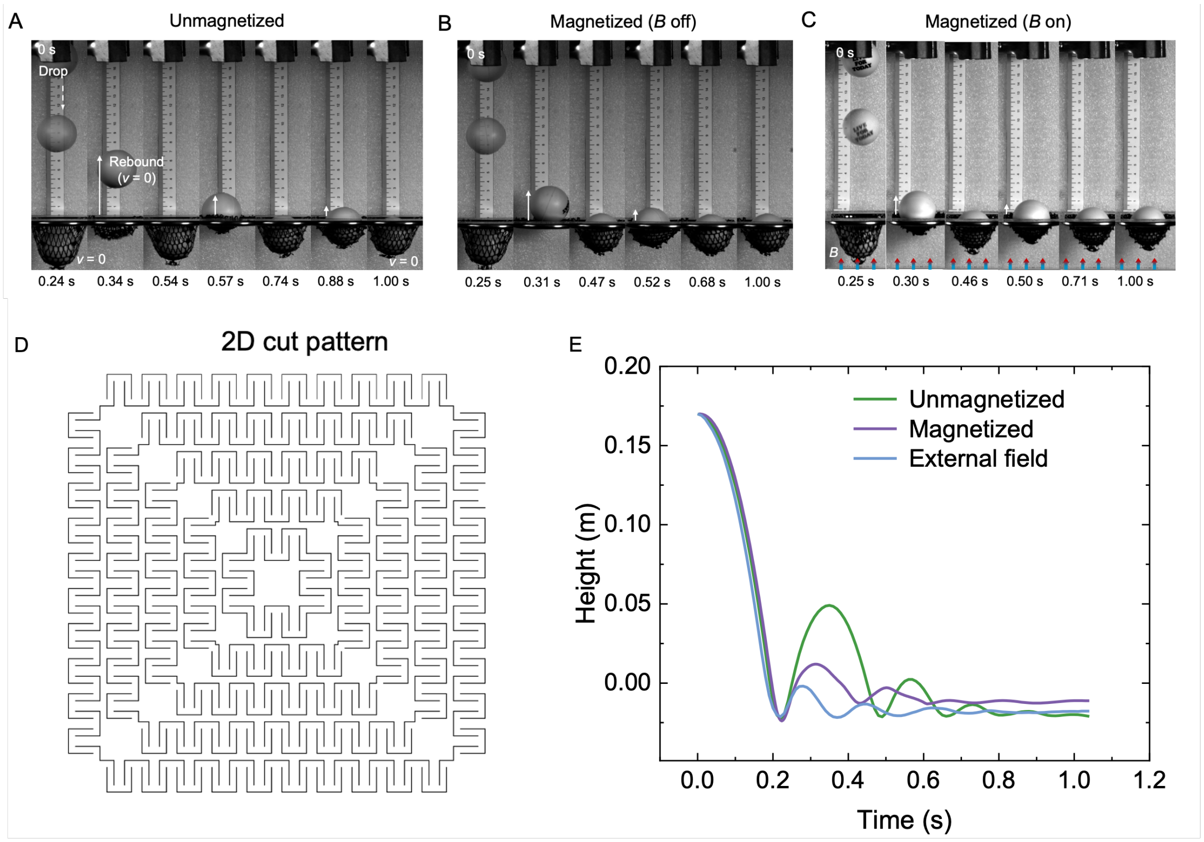

Fig. S12. Energy dissipation comparison between unmagnetized and magnetized single-layer mesh-like metamaterials with and without external magnetic field in the dropping test. (A-C) A small ball is released from a height of 0.15 m and interacts with the mesh in three conditions: non-magnetized and magnetized (without and with an applied external magnetic field *B*, 30 mT). The snapshots capture the moment when the ball's velocity reaches zero in each case. (D) Cut pattern used in the sample design. (E) Time-dependent height profiles of damped locomotion in the three cases shown in A–C. Compared to the unmagnetized case, the energy absorption increases by ~ 31% for the magnetized mesh with *B* off and 39% for the magnetized mesh with *B* on during the first impact.

Legends for Movies S1 to S11:

Movie S1: Front and side views of the uniaxial tension test on a single-layer magnetized metamaterial in a cycle of loading and unloading.

Movie S2: Front view of uniaxial tension test of a curved cut metamaterial.

Movie S3: Animated simulation of the tensile deformation process in a single magnetized metamaterial.

Movie S4: High-speed imaging of the sequential snapping process in a repulsive bilayer metamaterial.

Movie S5: Animated simulation of the two-step snapping process in a repulsive bilayer metamaterial.

Movie S6: Demonstration of stretching a repulsive bilayer metamaterial along varying directions.

Movie S7: High-speed imaging of the sequential snapping process in an attractive bilayer metamaterial.

Movie S8: High-speed imaging of the sequential snapping process in a trilayer metamaterial.

Movie S9: Comparison of the dropping ball test on a single-layer mesh using high-speed imaging for three cases: unmagnetized, magnetized, and with an applied external magnetic field.

Movie S10: Comparison of the dropping ball test on a bilayer mesh using high-speed imaging for three cases: unmagnetized, repulsive, and attractive.

Movie S11: Simultaneous buckling of the metamaterial induced by an external magnetic field at two distinct time points.
